# Patterns of conservation of spliceosomal intron structures and spliceosome divergence in representatives of the diplomonad and parabasalid lineages

**DOI:** 10.1186/s12862-019-1488-y

**Published:** 2019-08-02

**Authors:** Andrew J. Hudson, David C. McWatters, Bradley A. Bowser, Ashley N. Moore, Graham E. Larue, Scott W. Roy, Anthony G. Russell

**Affiliations:** 1 Alberta RNA Research and Training Institute, University of Lethbridge, Lethbridge, AB Canada; 2 Department of Biological Sciences, University of Lethbridge, Lethbridge, AB Canada; 3 Molecular Cell Biology, University of California-Merced, Merced, CA USA; 4 Department of Biology, San Francisco State University, San Francisco, California USA

**Keywords:** Spliceosomal intron, Small nuclear RNA, Spliceosomal protein, Intron evolution, Ancient intron, Diplomonad, *Spironucleus*, Ribosomal protein

## Abstract

**Background:**

Two spliceosomal intron types co-exist in eukaryotic precursor mRNAs and are excised by distinct U2-dependent and U12-dependent spliceosomes. In the diplomonad *Giardia lamblia*, small nuclear (sn) RNAs show hybrid characteristics of U2- and U12-dependent spliceosomal snRNAs and 5 of 11 identified remaining spliceosomal introns are *trans*-spliced. It is unknown whether unusual intron and spliceosome features are conserved in other diplomonads.

**Results:**

We have identified spliceosomal introns, snRNAs and proteins from two additional diplomonads for which genome information is currently available, *Spironucleus vortens* and *Spironucleus salmonicida*, as well as relatives, including 6 verified *cis-*spliceosomal introns in *S. vortens*. Intron splicing signals are mostly conserved between the *Spironucleus* species and *G. lamblia*. Similar to ‘long’ *G. lamblia* introns, RNA secondary structural potential is evident for ‘long’ (> 50 nt) *Spironucleus* introns as well as introns identified in the parabasalid *Trichomonas vaginalis*. Base pairing within these introns is predicted to constrain spatial distances between splice junctions to similar distances seen in the shorter and uniformly-sized introns in these organisms. We find that several remaining *Spironucleus* spliceosomal introns are ancient. We identified a candidate U2 snRNA from *S. vortens*, and U2 and U5 snRNAs in *S. salmonicida*; cumulatively, illustrating significant snRNA differences within some diplomonads. Finally, we studied spliceosomal protein complements and find protein sets in *Giardia*, *Spironucleus* and *Trepomonas* sp. PC1 highly- reduced but well conserved across the clade, with between 44 and 62 out of 174 studied spliceosomal proteins detectable. Comparison with more distant relatives revealed a highly nested pattern, with the more intron-rich fornicate *Kipferlia bialata* retaining 87 total proteins including nearly all those observed in the diplomonad representatives, and the oxymonad *Monocercomonoides* retaining 115 total proteins including nearly all those observed in *K. bialata*.

**Conclusions:**

Comparisons in diplomonad representatives and species of other closely-related metamonad groups indicates similar patterns of intron structural conservation and spliceosomal protein composition but significant divergence of snRNA structure in genomically-reduced species. Relative to other eukaryotes, loss of evolutionarily-conserved snRNA domains and common sets of spliceosomal proteins point to a more streamlined splicing mechanism, where intron sequences and structures may be functionally compensating for the minimalization of spliceosome components*.*

**Electronic supplementary material:**

The online version of this article (10.1186/s12862-019-1488-y) contains supplementary material, which is available to authorized users.

## Background

Eukaryotic nuclear genomes contain spliceosomal introns which divide protein-coding sequences into separate exons. Exons must then be ligated together during precursor mRNA splicing, before mRNA transit to the cytoplasm for protein translation. Spliceosomal introns have been identified in virtually all eukaryotes, however, intron density is remarkably variable across species. Some intron-poor species contain only a few introns per genome [1, 2] while some intron-rich species have on average several introns per kilobase of gene sequence [3]. Intron length may also differ substantially, from introns as short as 15 nt in the ciliate *Stentor coeruleus* [4] to mammalian gene introns which can reach many tens of kilobases in size [3]. A few previous studies have also revealed phylogenetic diversity of the spliceosomal machinery itself, however less is known about the evolution of the spliceosome than about the evolution of introns.

Thus far, two separate classes of spliceosomal introns have been identified in eukaryotes: the major/U2-type and minor/U12-type spliceosomal introns. U2-type introns have been identified in nearly all fully-sequenced nuclear genomes, whereas only a subset of eukaryotes have been found to contain U12-type introns [5, 6]. However, the distribution of U12-type introns in evolutionarily-diverse eukaryotes reveals an ancient origin for U12-type introns and indicates they were very likely present in the last eukaryotic common ancestor [5, 6].

Removal of U2-type introns is catalyzed by the major/U2-dependent spliceosome consisting of the five evolutionarily conserved small nuclear RNAs (snRNAs) U1, U2, U4, U5 and U6 and dozens to several hundred spliceosomal proteins [7]. U12-type introns are excised by a distinct minor/U12-type spliceosome that contains both shared U2-dependent and unique U12-dependent spliceosomal proteins, the common U5 snRNA and uniquely the U11, U12, U4atac and U6atac snRNAs which are functionally analogous to the U1, U2, U4 and U6 snRNAs, respectively [8]. U2- or U12-type spliceosomal introns are distinguished by distinctive 5′ and 3′ splice sites (SS) and internal branch point (BP) sequence motifs that are recognized in part via specific RNA-RNA base pairing interactions with U2- or U12-dependent spliceosomal snRNAs [9].

These core non-coding snRNAs are joined by up to hundreds of associated proteins to give rise to the highly dynamic, megadalton sized spliceosome. The spliceosome is generally highly conserved, with components and subcomplexes being shared across deeply diverged eukaryotes [7, 10]. For instance, among well-studied organisms, the spliceosomes of *H. sapiens*, *A. thaliana*, and *S. cerevisiae* all contain generally conserved core components and subcomplexes. Previous studies have probed the spliceosome in eukaryotes with highly-reduced intron numbers, and interestingly have found correspondingly reduced spliceosomes. For instance, a recent study of *C. merolae* found only around 40 spliceosomal proteins among the general eukaryotic core of > 100, and revealed complete loss of the entire U1 snRNP subcomplex [11, 12]. Previous results suggest similar transformation in other lineages [11–13]. Such a correspondence may even hold among model organisms: *S. cerevisiae*, in which only ~ 5% of genes contain introns, also has the fewest conserved spliceosomal components of well-studied models [13].

While some spliceosomal introns are only positionally-conserved in closely-related taxa, many are conserved over very long evolutionary distances. For instance, ~ 25% of introns in *Arabidopsis thaliana* occupy the same position in orthologous genes in humans [14] and some of the introns in intron-reduced protist species show conservation in distantly-related, intron-rich species [15, 16]. For example, the *Rpl7a* gene intron in *Giardia lamblia* (syn *G. intestinalis*) is also present in orthologous genes in animals and some Amoebozoans [16]. Reconstruction of ancestral eukaryotic intron density based on patterns of intron gain and loss in 99 different eukaryotes suggests that the last eukaryotic common ancestor (LECA) was intron-rich [17] and already endowed with a complex spliceosomal apparatus [13]. The identification of ancient spliceosomal introns with functions conserved in diverse eukaryotes would indicate very early beneficial intron function.

Diplomonads are a group of eukaryotes with characterized species containing highly-reduced nuclear genomes and apparently few spliceosomal introns [1, 18]. The first characterized spliceosomal introns in diplomonads were identified in *G. lamblia*. These introns contain extended highly-conserved 5′ splice site sequences, with the BP fused to the 3′ SS sequences and a high proportion of introns that are spliced in *trans* from two or more precursor mRNAs [16, 19–21]. *Trichomonas vaginalis*, a parabasalid (diplomonad sister group), shares the same general spliceosomal intron structure and splice site sequence motifs as *G. lamblia* [15] and a priori, one would therefore predict that other diplomonads will share these conserved intron features.

In this study, we used bioinformatics to identify the spliceosomal machinery and introns of additional diplomonad species. We first identified a set of introns in *Spironucleus vortens* by specifically examining ribosomal protein (RP) genes which then allowed us to design search parameters to identify additional introns in this organism. We then used 5′ RACE experiments to examine the removal of the predicted introns. Intron splicing consensus sequences in the *Spironucleus* introns then provided information for the bioinformatic prediction of U2 and U5 snRNAs by analysis of *S. vortens* and *S. salmonicida* genomic sequences. We find striking conservation of intron structural properties in the examined diplomonads and a parabasalid and observe that many of the spliceosomal introns in *Spironucleus* are ancient introns found conserved in RP genes. We also searched the proteomes of *G. lamblia, S. salmonicida,* and *Trepomonas* sp. PC1, as well as related fornicate and oxymonad protists, providing a portrait into mechanisms of splicing in these highly reduced organisms as well as insights into the dynamics of spliceosomal reduction.

## Results

### Spliceosomal introns in RP and non-RP genes in *S. vortens*

Only 11 spliceosomal introns have been identified in the diplomonad *Giardia lamblia* [1, 16, 19–23], revealing both a remarkable paucity of spliceosomal introns and also proportionally large number of *trans-*spliced introns in this organism. Despite this, little is known about spliceosomal intron structure from members of the diplomonad genus *Spironucleus*. More recently, sequencing the genome of *S. salmonicida* uncovered three experimentally-confirmed *cis*-spliceosomal introns [18]. To further expand our knowledge of spliceosomal intron structure in diplomonads, we searched the preliminary nuclear genomic DNA sequence data from *Spironucleus vortens* for spliceosomal introns. Initially our search strategy employed the conserved *G. lamblia* 5′ splice site (SS) sequence ‘VTATGTT’ and fused branch point (BP) and 3′ SS sequence ‘VCTRACACRCAG’ (‘R’ is a purine; ‘V’ is an A, C, or G nucleotide) [20], but these searches did not identify any introns in *S. vortens*. Thus, we reasoned that intron splice site sequences differ in *S. vortens* compared to *G. lamblia* and *T. vaginalis* introns.

**Fig. 1:**
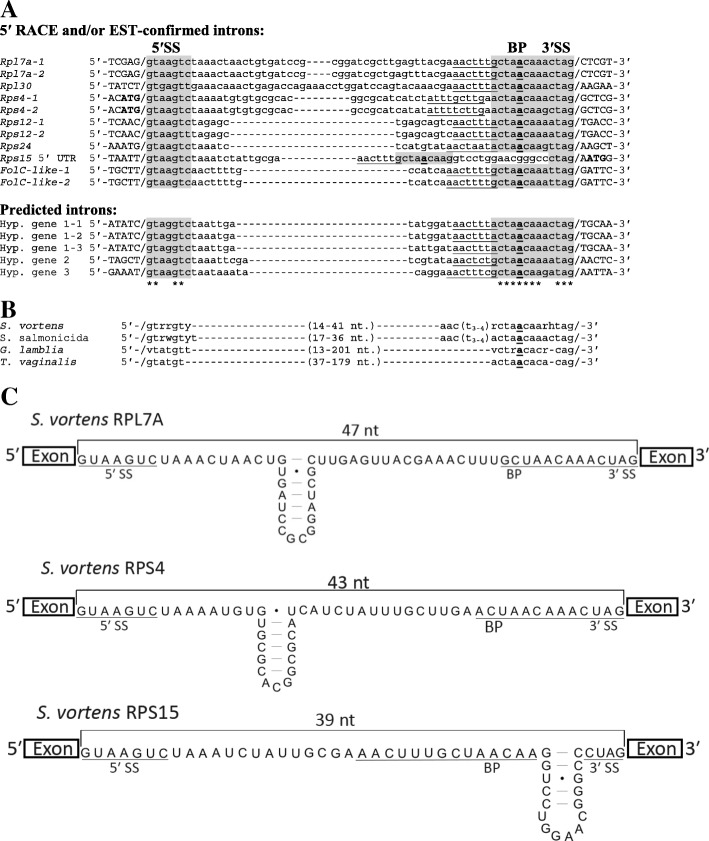
*Cis-s*pliceosomal introns in *S. vortens*. (**a**, **b**) 5′ RACE and/or EST-confirmed (RP and *FolC-like* genes) and predicted (Hypothetical genes) spliceosomal intron sequences from *S. vortens* genomic sequences were aligned using ClustalW2 software (see Additional file 2 for gene sequences and EST accession numbers). The 5 nt of exonic sequences flanking each intron are in upper case with a slash representing the exon-intron boundary and mRNA start ‘ATG’ codons in bold. Predicted intron 5′ and 3′ splice sites (SS) and branch point (BP) sequences are highlighted in grey with the putative reactive branch point adenosine in bold and underlined. A conserved pyrimidine-rich motif (‘AAC [T/C]_3-4_R’) found upstream of the branch point sequence is underlined. Nucleotide identities shared between all aligned introns are indicated by asterisks under the alignment. **b** Consensus sequences from the identified *S. vortens* spliceosomal introns are compared to those from the related diplomonad *Spironucleus salmonicida* [18], *Giardia lamblia* [20] and parabasalid *Trichomonas vaginalis* [15]. An ‘R’ indicates a purine, ‘Y’ is a pyrimidine, ‘W’ is A/T, ‘V’ is A/C/G and ‘H’ is A/C/T. **c** Secondary structural potential for *S. vortens* long *cis*-introns with predicted internal stem loops (See Fig. 3 for *Rpl30*). Predicted 5′/3′ SS and BP motifs are underlined. Lengths of ‘single-stranded’ distances between splice donor and acceptor sites are indicated in nucleotides (nt) above the intron sequences

**Fig. 2:**
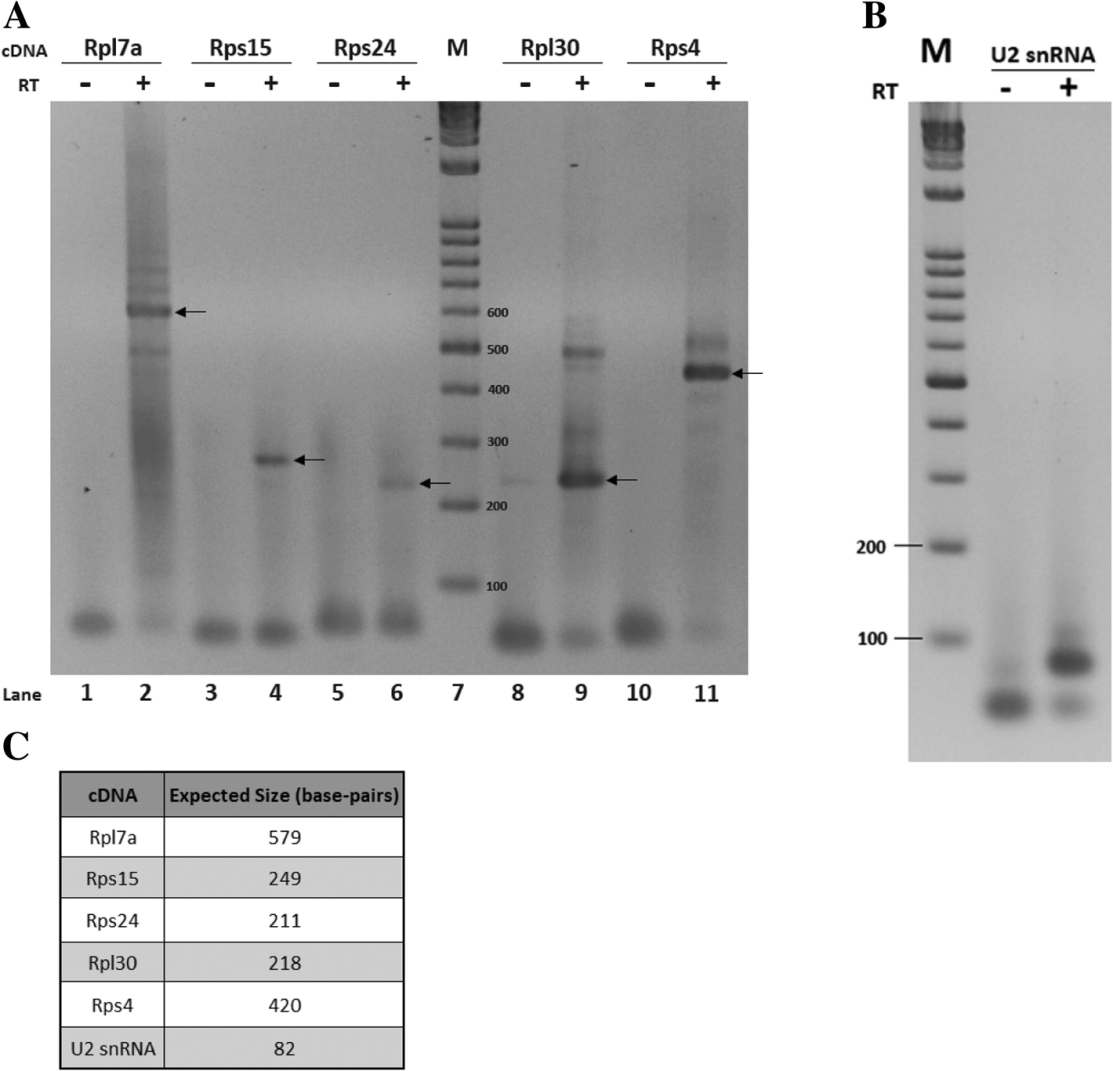
Confirmation of ribosomal protein mRNA intron removal by 5′ RACE, and RT-PCR verification of U2 snRNA expression. **a** 5′ RACE analysis of spliced mRNAs expressed from predicted intron-containing ribosomal protein (RP) genes or **b**) RT-PCR detection of expression of the candidate *S. vortens* U2 snRNA. Products have been resolved on 3% agarose gels. Arrows indicate the bands of expected size for cDNA products indicative of intron removal. Inclusion of reverse transcriptase (+) or its omission (−) during the reactions are indicated above each lane. Lanes labeled M contain a molecular weight DNA size ladder with band sizes indicated in base pairs. **c** Table indicating expected sizes for cDNA products from the RP mRNAs following intron removal based on predicted or EST-confirmed 5′ end, and also for the RT-PCR product for the targeted U2 snRNA segment

Ribosomal protein (RP) genes are highly-conserved protein-coding sequences readily recognizable in eukaryotic genomes. Notably, some intron-poor eukaryotes (e.g. *S. cerevisiae* and the microsporidian *Encephalitozoon cuniculi* (PMID: 20360213) contain a large proportion of their spliceosomal introns within RP genes [24, 25] and one of the few *cis*-spliced introns in *G. lamblia* interrupts the *Rpl7a* gene [16]. Therefore, we determined whether protein-coding continuity in RP genes is interrupted by one or more spliceosomal introns in *S. vortens*. RP genes have not been previously annotated in *S. vortens*, so we initially performed homology searches using the 80 RP genes from *Saccharomyces cerevisiae* [26] as queries for TBLASTN searches against the *S. vortens* raw genomic sequence data. These searches identified 70 predicted RP gene sequences in *S. vortens* (data not shown). Next, the *S. vortens* RP gene sequences were individually aligned with corresponding expressed sequence tag (EST) data to determine if they contained intervening sequences not present in mature mRNAs. This analysis identified single spliceosomal introns interrupting conserved regions of RP genes *Rpl7a*, *Rpl30*, *Rps4*, *Rps12* and *Rps24* (Fig. 1a and Additional files 2 and 3). In each case, intron sequences contain an in-frame stop codon and/or introduced a frame shift (in the downstream coding region) that would result in a truncated ribosomal protein (Additional file 3). With the exception of *Rps12*, we were able to further confirm intron removal for each RP gene and map their 5′ mRNA ends using 5′ RACE (Fig. 2a). We also observed RP gene sequence variants during the analysis that appear to be allelic variants (*S. vortens* is tetraploid), based on the high-level of nucleotide sequence similarity (Additional file 4) and identical chromosomal context. Allelic variants of the *Rpl7a*, *Rps4* and *Rps12* genes contained intron sequence differences and thus were included in the subsequent intron analyses (Fig. 1a and Additional file 4).

Our non-sequence biased identification of introns in *S. vortens* RP genes revealed a consensus sequence ‘GTAAGTY’ at the 5′ SS, and a branch point fused to 3′ SS sequence, ‘RCTAACAARHTAG’ (predicted BP ‘A’ is underlined, ‘R’ is purine, ‘Y’ is pyrimidine, ‘H’ is A, C or T) (Fig. 1). Using these identified conserved intron sequence features, we next searched for additional introns in genomic sequences by employing the sequence pattern matching program ‘Scan for Matches’ [27]. This search strategy uncovered an additional EST-verified intron interrupting a ‘bifunctional folylpolyglutamate synthase-like’ (*FolC-like*) gene and three additional putative introns individually interrupting three different predicted protein-coding genes of unknown function (Fig. 1a and Additional file 2). Although intron insertion sites may be located outside of protein-coding sequences, it should be noted that *S. vortens* only utilizes one stop codon in its genetic code [28], increasing the likelihood of inaccurate protein-coding gene prediction. In the absence of both EST and RT-PCR data to confirm gene expression and due to the lack of open-reading frame (ORF) conservation in other characterized species, these three additional putative introns will require further experimental verification.

The *S. vortens* spliceosomal introns collectively are short and relatively uniform in size, ranging from 40 to 67 nucleotides, and are primarily located proximal to the 5′ ends of the ORFs. The *Rps4* intron is located immediately downstream of the ‘ATG’ start codon, a so-called ‘start codon intron’ – a location often observed in the RP gene sequences of other eukaryotes [29]. We also note that most *S. vortens* introns are phase ‘0’ introns (5 of the 6 confirmed introns).

The *S. vortens* intron sequences display extended sequence conservation of intron splice sites. In addition to standard ‘GT-AG’ boundaries, the introns display a 7 nt conserved 5′ SS and 13 nt fused BP + 3′ SS sequence, akin to the spliceosomal introns in *G. lamblia* and those identified in *S. salmonicida* (Fig. 1b). Interestingly, we also note that some of the *S. vortens* introns display substantial sequence similarity to each other in the internal region between the 5′ SS and BP + 3′ SS. For example, in an optimized alignment of the *Rpl7a* and *Rpl30* introns there is ~ 70% nucleotide identity over the entire intron length (44 out of 63 nt positions), and ~ 63% identity (27 of 43 positions) when excluding the 5′ SS and BP + 3′ SS sequence elements from the comparison (Fig. 1a). Furthermore, 3 of 5 RP gene introns and 2 of the introns in the putative protein-coding genes contain the sequence ‘TAAA’ starting at intron position + 8 which would extend the 5′ SS consensus to ‘GTARGTYTAAA’ for these introns. Also evident is a recurring pyrimidine tract-containing sequence motif, directly upstream adjacent to the intron branch point sequence, with consensus sequence ‘AAC [T/C]_3-4_R’ (Fig. 1a, underlined). The *FolC-like* gene intron contains an additional copy of this motif downstream adjacent to its 5′ SS sequence (Fig. 1a). Notably, the three confirmed *S. salmonicida* introns [18] also display the ‘AAC [T/C]_3-4_R’ motif sequence and a similar A-T extended 5′ splice site motif ‘GTATGTTTAAC.’

### A 5′ UTR intron in the *S. vortens Rps15* gene

Based on intron sequence conservation, we also identified an intron-like sequence in the 5′ UTR region of the *Rps15* gene through a BLASTN search using the newly-identified *Rpl7a* intron as query. The sequence was a plausible intron candidate due to the presence of a canonical and extended 5′ SS sequence ‘GTAAGTCTAAA’, BP and pyrimidine-tract (underlined) motif sequence ‘AACTTTGCTAACAA’ (Fig. 1a), as found in the *Rpl7a* intron (Fig. 1a and c). However, unlike the other *S. vortens* introns, the candidate *Rps15* intron’s 3′ SS sequence motif ‘CTAG’ is not fused to the BP sequence and instead is displaced downstream by 15 nt. The distance between the BP ‘A’ and 3′ SS is a highly conserved property of all identified *G. lamblia* introns [16, 20]. Experiments in *T. vaginalis* in which the BP motif (‘ACTAAC’) was moved 2 or 7 nt upstream of its conserved position abolished splicing in an in vivo assay [15] indicating a requirement for the precise spacing of these intron elements in the splicing reaction mechanism in these organisms. Based on this, one might initially predict that the ‘inserted’ sequence in the *Rps15* intron-like element may prevent splicing of this region.

Closer examination of this insertion sequence reveals an inverted repeat that could form an RNA stem-loop element containing 5 consecutive base pairs (italicized sequence in Fig. 1a and c) in the mRNA transcript. This would bring the BP and 3′ SS-like sequence into closer spatial proximity and suggests the alternative possibility of a functional role of the stem-loop element in splicing of this *Rps15* intron. To determine if the *Rps15* UTR intron is removed in mature mRNA, we performed 5′ RACE. Remarkably, we observed a single 5′ RACE product whose size corresponded to that of a spliced *Rps15* mRNA lacking the 5′ UTR intron (Fig. 2a). Sequencing of the 5′ RACE product confirmed intron removal had occurred at the predicated 5′ and 3′ splice sites with the mapped 5′ end of the mature mRNA only 12 nt upstream of the 5′ SS (Additional file 7). This suggests that the formation of a stem-loop element in the *Rps15* intron sufficiently reduces the distance between the BP and 3′ SS to allow for efficient intron removal, consistent with the other identified *S. vortens* introns. It is also possible, although seemingly less likely, that *S. vortens* is more flexible in its requirements for proximity of the BP and 3′ SS during splicing compared to *G. lamblia* and *T. vaginalis* and the splicing mechanism tolerates the additional inserted nucleotides without the need for stem-loop formation.

### Genome-wide search in *S. salmonicida* reveals no new introns

We also leveraged available full-genome and RNA-Seq data from *S. salmonicida* to attempt to identify previously unreported introns. Mapping RNA-Seq data to the genome with permissive criteria identified 10,153 candidate sequences with either canonical or noncanonical splice boundaries. The vast majority of these are likely to represent sequencing artifacts. In particular, most showed the signature of template switching by reverse transcriptase during cDNA preparation (49.8% of candidates had a perfect five-nucleotide in-frame match within 5 nucleotides of the boundaries, and an additional 22.9% had a 4/5 match). We further manually studied all candidates with (i) a near match to observed 5′ consensus splice boundaries (5/6 matches to GTATGT or to GTGAGT); (ii) ending in [CT]AG; or (iii) having a candidate branchpoint ACT [AG] AC within 20 nucleotides of the 3′ end. No clear novel candidates were identified.

### Base pairing potential in *S. vortens* and *T. vaginalis* introns

**Fig. 3:**
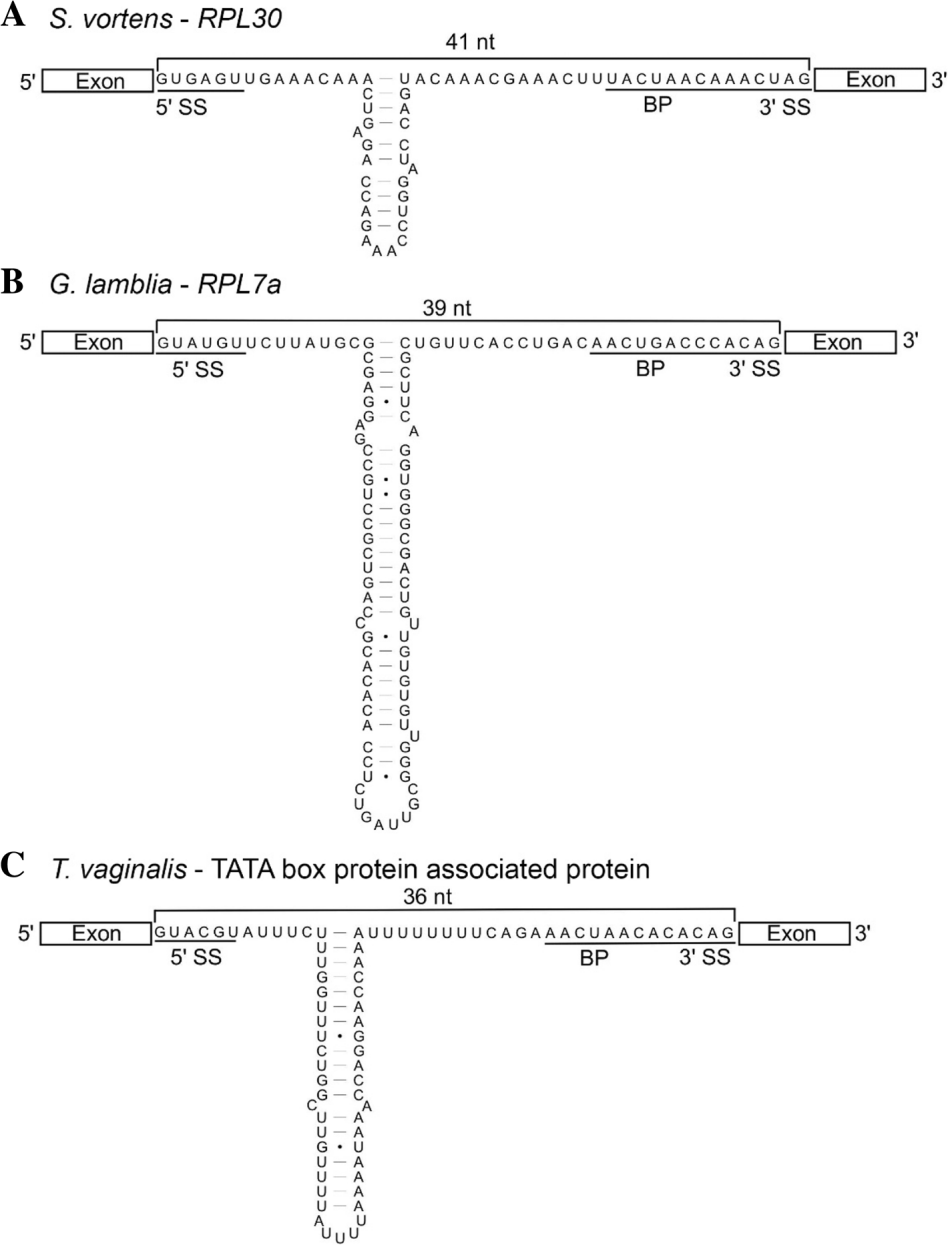
Base pairing within long *cis*-spliced introns in diplomonads and a parabasalid. Secondary structural predictions of representative *cis*-spliceosomal introns from *S. vortens* (**a**), *G. lamblia* (**b**), and *T. vaginalis* (**c**) are shown with putative 5′/3′ splice site (SS) and branch point (BP) motifs underlined. Lengths of ‘single-stranded’ distances between splice donor and acceptor sites are indicated in nucleotides (nt) above the intron sequences

The collection of ‘long’ *cis*- and *trans-*spliced introns in *G. lamblia* display extensive secondary structural potential which appears to constrain the spatial distance between splice donor and acceptor sites to 35–45 nt – a similar length to the characterized short *G. lamblia cis*-spliced introns (Fig. 3b) [20]. Therefore, we examined *S. vortens* introns for similar internal base pairing potential. Intriguingly, while the length of the ‘short’ *S. vortens* introns cluster uniformly at 40–42 nt, MFOLD secondary structure predictions indicate that the longer *Rps15, Rpl7a*, *Rpl30* and *Rps4* introns may form stable stem-loop elements, thus bringing the splice sites within similar spatial proximity (Figs. 1c and 3). We also found that the ‘long’ *S. salmonicida Rpl30* intron [18] is capable of forming a stem-loop element, making its splice donor to splice acceptor spatial length 41 nt, similar to the total length of the other short *S. salmonicida* introns (43 nt) (Additional file 5).

Stimulated by our discovery of structural potential in *Spironucleus* introns, we next examined secondary structural potential in *T. vaginalis* introns to assess whether similar intron internal base pairing potential is a more wide-spread phenomenon. We found that most *T. vaginalis* introns were either uniformly short (~ 25 nt) or else were longer (> 50 nt) but with the ability to form extended stem-loop elements, making intron splice boundary spatial lengths between 25 and 44 nt (median of 37 nt) upon intron folding (Fig. 3c and Additional file 6).

### The phylogenetic distribution of the *Rps4* and *Rps24* introns indicates they are ancient introns

**Fig. 4:**
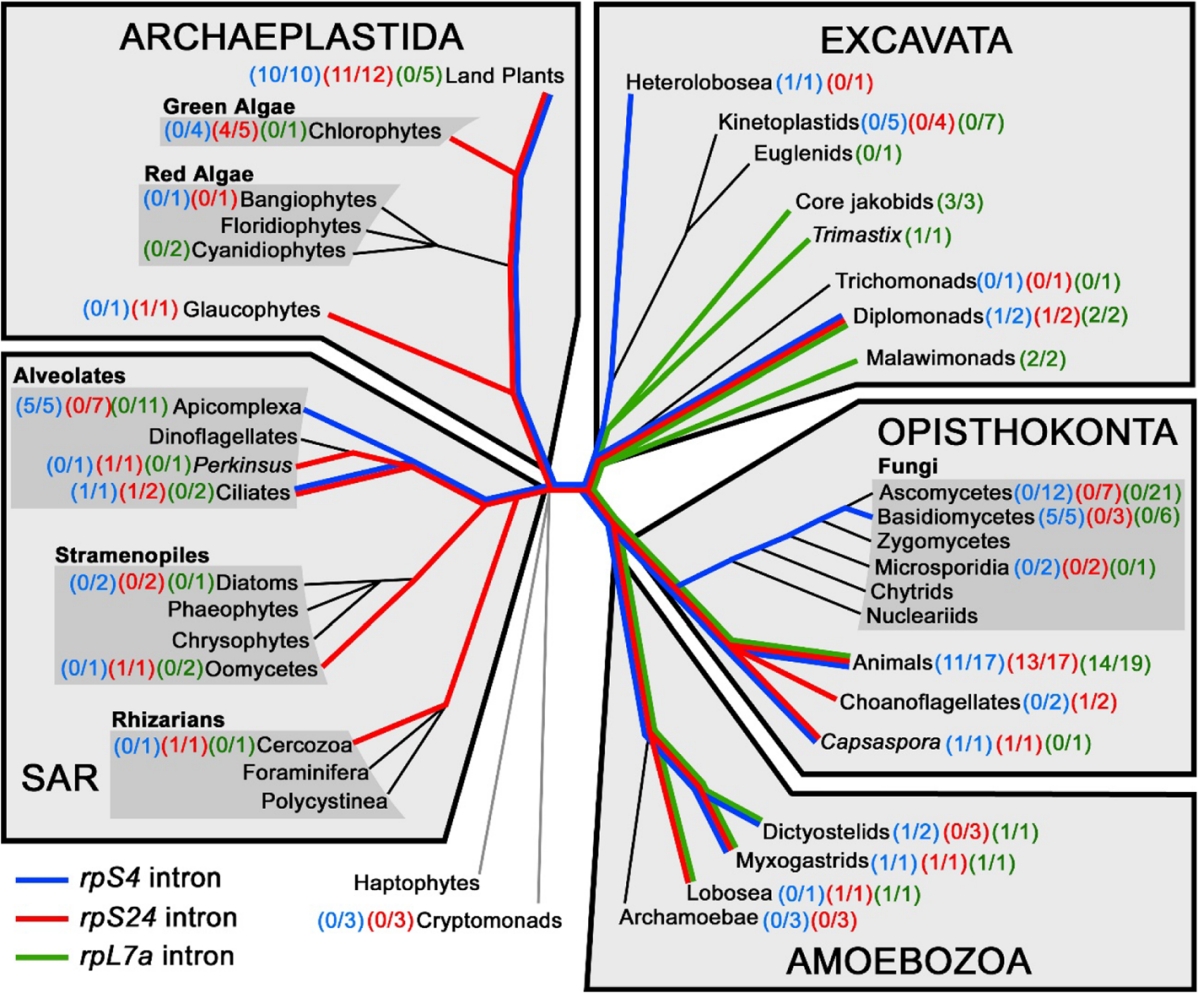
Phylogenetic distribution of RP gene introns in eukaryotes. Representative eukaryotes from each eukaryotic supergroup were examined for intron insertion at the same conserved position within *Rps4* (blue), *Rps24* (red) and *Rpl7a* (green) [16] genes and the distribution for each intron was mapped onto a eukaryotic tree by Burki (2014) [30]. The number of species containing an intron (numerator) and the number sampled (denominator) are indicated for each eukaryotic group (See Additional file 8 for organism names). Coloured lines indicate extant eukaryotic groups which contain each intron to the predicted last common ancestor to contain each respective RP gene intron

The previous examination of the *G. lamblia Rpl7a* intron revealed intron conservation at the identical position within *Rpl7a* orthologs from representative organisms of two of the five currently accepted eukaryotic supergroups [16] (Fig. 4). This phylogenetic conservation of the *Rpl7a* intron indicated an early creation of this intron in eukaryotic evolution. Polymerase chain reaction (PCR) analysis of *Spironucleus barkhanus* genomic DNA indicated a lack of the *Rpl7a* intron in this species [16]. We have now discovered that *Spironucleus vortens* contains the *Rpl7a* intron (Fig. 1a and Additional file 7) indicating recent loss of the *Rpl7a* intron in some *Spironucleus* species.

**Fig. 5:**
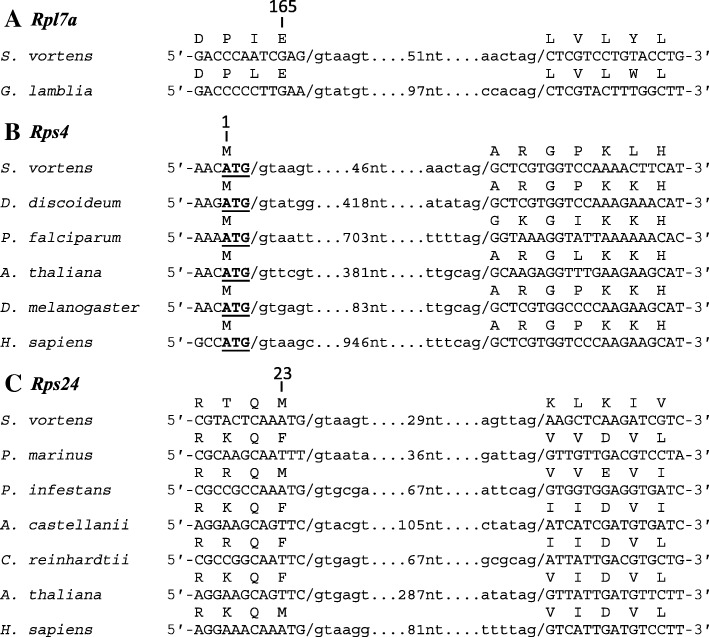
Conservation of *Rpl7a*, *Rps4* and *Rps24* intron insertion sites. Gene sequences from representative eukaryotes containing *Rpl7a* (**a**), *Rps4* (**b**) and *Rps24* (**c**) spliceosomal introns are aligned with slashes (/) representing intron-exon boundaries; intronic sequences in lower case and exonic sequences in uppercase. The number of nucleotides between splice site sequences is indicated. Translated amino acid sequences are shown above the first nucleotide of each codon and the start ‘ATG’ codons for the *Rps4* coding sequences are underlined. Amino acid positions for each protein are indicated based on the *H. sapiens* orthologs. NCBI accession numbers for (**a**) *Rpl7a* - *S. vortens* [NCBI Trace Archive:ti|2,141,515,448], *G. lamblia* [GenBank:NW_002477099], **b** *Rps4* - *S. vortens* [ti|2,141,550,682], *D. discoideum* [NC_007088], *P. falciparum* [NC_004315], *A. thaliana* [NC_003071], *D. melanogaster* [NT_037436] and *H. sapiens* [NC_000023] and (**c**) *Rps24* – *S. vortens* [ti|2,141,541,737], *P. marinus* [NW_003201404], *P. infestans* [NW_003303749], *A. castellanii* [NW_004457654], *C. reinhardtii* [NW_001843791], *A. thaliana* [NC_003074] and *H. sapiens* [NC_000010]

We next analyzed the conservation patterns of the other *S. vortens* RP gene introns. We examined more than 80 eukaryotes representing all five proposed eukaryotic supergroups [30] and identified spliceosomal introns in identical position and phase in the *Rps4* and *Rps24* genes in several other distantly-related organisms (Fig. 5b and c). For both genes, at least one representative organism within each of the five supergroups contains an intron at the same position as *S. vortens* with some organisms conserving both introns; the *Rps24* intron displays a somewhat wider distribution (Fig. 4 and Additional file 8). The introns are nearly always inserted at the same relative coding position and phase within the ORF; however, we also found some potential evidence of ‘intron sliding’ in which organisms had an *Rps4* or *Rsp24* intron in an adjacent codon to the conserved intron insertion position (data not shown). Collectively, our analyses reveal that the *Rps4* and *Rps24* introns may be even more widespread than the *Rpl7a* intron (Fig. 4).

We also found spliceosomal introns in the *S. vortens Rpl30* and *Rps12* genes in the same relative positions as those in humans. These are found in less well-conserved regions of RP amino acid sequence and therefore it is more difficult to ascertain whether these represent ancient intron insertion events or more recent independent intron acquisitions in nearby sites.

An alternative (but less parsimonious) explanation for the observed evolutionary distribution of the *Rps4* and *Rps24* introns was the occurrence of numerous independent and widespread intron gain events at proto-splice sites in these genes. Nucleotide sequence in the flanking exon portions adjacent to either the *Rps4* or *Rps24* introns shows conservation amongst distantly-related eukaryotes, consistent with conserved RP amino acid sequence encoded by these regions. For the *Rps24* intron, these sequences do not conform to the proto-splice site consensus (A/C)AG/G [31]. However, the exonic sequences flanking the *Rps4* intron are a better match (typically 3 out of 4 nt). Because exonic sequence encodes the invariant ‘ATG’ start codon (proto-splice site nucleotides underlined) and conserved alanine (‘GCN’) or glycine (‘GGN’) resides, we cannot refute the possibility that the widespread distribution of the *Rps4* intron is the result of multiple independent intron gain events. Thus, we conclude that the observed distribution of the *Rpl7a* and *Rps24* introns are not likely due to independent intron gains at proto-splice sites and the phylogenetic distribution of *Rps24* introns may be explained by single ancient intron gain events in the last common ancestor of the examined taxa.

### Identification of *Spironucleus* spliceosomal snRNAs

**Fig. 6:**
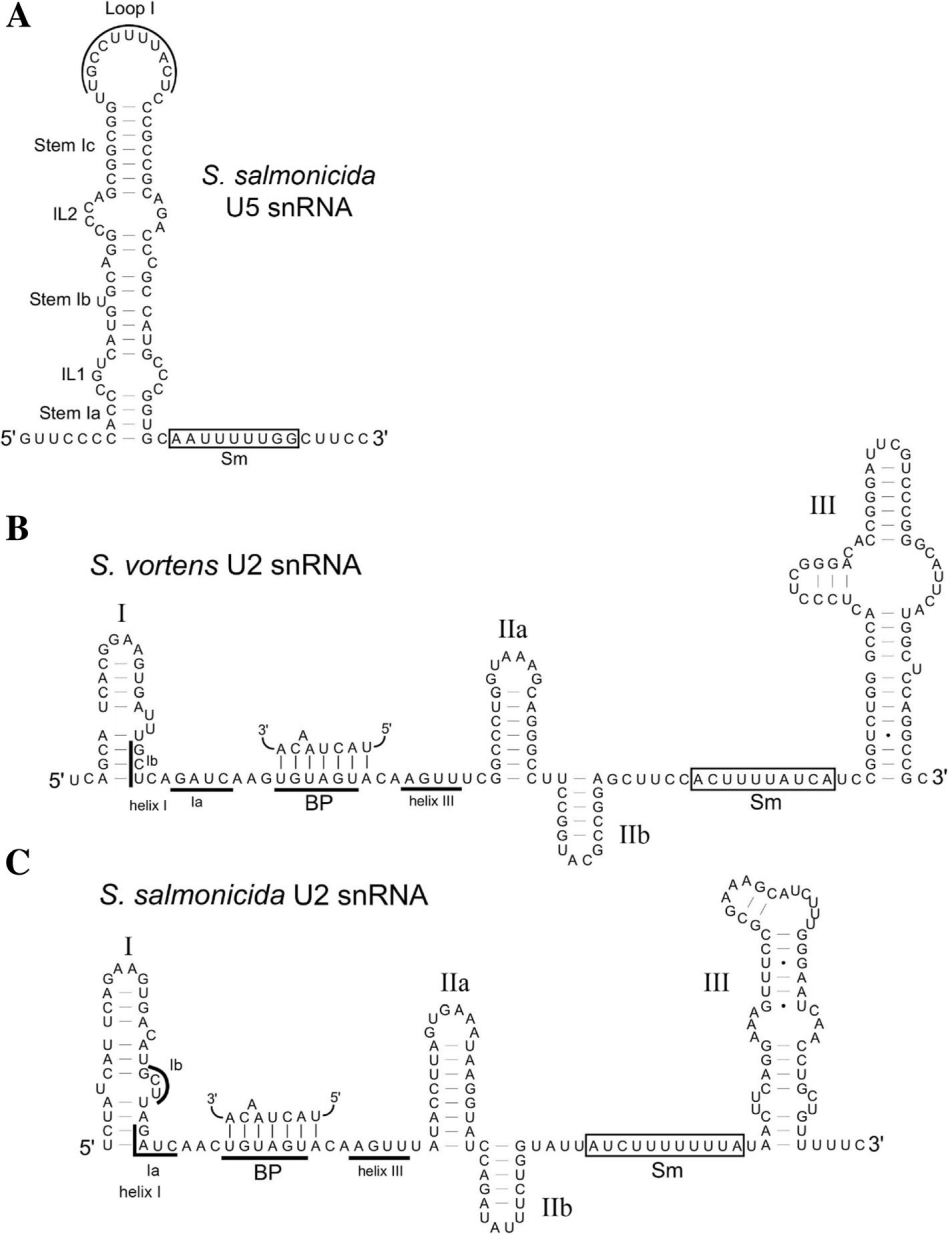
Spliceosomal snRNAs from *S. vortens* and *S. salmonicida*. **a**-**c** Predicted secondary structures (MFOLD) for *Spironucleus* snRNA sequences are shown with conserved sequence and structural elements indicated. Sm = Sm protein binding site. **b**, **c** U2 snRNAs in *Spironucleus*. BP = branch point interacting sequence. Helix I and III are regions of U2 snRNA predicted to form intermolecular base pairs with U6 snRNA. Accession numbers of genomic contigs containing snRNA sequences are: **a** *S. salmonicida* U5 (GenBank AUWU01000115:5304–5391), **b** *S. vortens* U2 (ti|2,141,663,608:84–246) and (**c**) *S. salmonicida* U2 (AUWU01000434:68649–68,502)

During pre-mRNA splicing, intron substrates are recognized by the spliceosome in part by RNA-RNA intermolecular base pairing involving U1, U2, and U6 snRNAs with intron 5′ SS and BP sequences [9]. Initially, we had performed BLASTN searches using the *G. lamblia* U1, U2, U4 and U6 snRNAs [32] as queries against *S. vortens* and *S. salmonicida* genomic sequences; however, these searches did not yield any plausible snRNA candidates. Co-variation models (CMs) are probabilistic models that combine consensus RNA sequence and secondary structures from a known RNA family to search for probable RNA species in a DNA/RNA database. Moreover, CMs have been successfully employed to predict snRNA-like sequences in genomic DNA sequences from diverse eukaryotes [33]. Thus, we performed CM searches for all major and minor-spliceosomal snRNA-like sequences in *Spironucleus* DNA sequences using the Infernal software package [34] and CMs generated with U-snRNA sequences from the Rfam database [35]. It was necessary to generate new CMs (rather than those stored in Rfam) using select snRNA sequences from distantly-related eukaryotes to more heavily weight the most conserved snRNA features and therefore increase the likelihood of identifying candidates in organisms containing highly-diverged snRNAs (as previously observed in *G. lamblia*). These searches identified convincing U2 snRNA candidates for *S. vortens* and *S. salmonicida* (Fig. 6); however, they were not successful in identifying plausible candidates of other major nor any minor (U12-type) spliceosomal snRNAs.

Examination of the *S. vortens* and *S. salmonicida* U2 snRNA candidates reveals secondary structural features of U2 snRNAs from other representative eukaryotes, with identifiable SLs I, IIa/IIb and III and predicted Sm protein binding sites (Fig. 6b and c). However, the SL IV found in most other U2 snRNAs [8] appears to be missing in both the *S. vortens* and *S. salmonicida* U2 candidates. The 5′ half of both *Spironucleus* U2 candidates contain branch point-interacting sequences that would generate the expected bulged intronic catalytic adenosine upon base pairing interaction (Fig. 6b and c). We also note that the conserved U2 snRNA ‘GCU’ and ‘GAUC’ sequences involved in formation of U2-U6 intermolecular helix I [36] are conserved in both *Spironucleus* U2 candidates. Furthermore, while the first ~ 45 nt of the *S. vortens* U2 snRNA candidate displays high sequence conservation to the *S. salmonicida* U2 candidate (36/45 nucleotide identity), the remaining downstream sequences are divergent, yet both maintain the ability to form structurally-conserved SL IIa/IIb and an extended SL III. To validate expression of the putative *S. vortens* U2 candidate, we performed RT-PCR on *S. vortens* total RNA using U2 candidate-specific primers. RT-PCR produced a single product of expected size (Fig. 2b) and subsequent DNA sequencing confirmed successful amplification of the U2 snRNA fragment (Additional file 7).

CM searches did not identify any plausible U5 snRNA candidates. However, U5 snRNAs are typified by a long stem-loop containing the highly-conserved loop I sequence ‘UGCCUUUUACY’ involved in binding exons during the splicing reaction [37]. Therefore, we reasoned that ‘Scan for Matches’ may be more successful in finding U5 snRNA-like sequences in *Spironucleus spp.* DNA sequences by searching for instances of the canonical loop I sequence motif or variants thereof (allowing 2 substitutions), flanked by sequences capable of forming a 6 bp apical stem Ic expected in U5 snRNA structures. One pattern match in *S. salmonicida* displayed a perfect loop I sequence match (UGCCUUUUACU) and upon closer examination, was capable of not only forming stem Ic, but also a canonical extended SL I containing internal loops (IL) 1 and 2 followed by a predicted Sm protein binding site (Fig. 6a). So far, this strategy has not identified obvious U5 snRNA-like sequences in *S. vortens* (see Discussion).

### Greatly reduced complement of spliceosomal proteins in *Spironucleus* and relatives

As observations of other organisms with reduced intron density have revealed protein component loss of the spliceosomal machinery, we performed bioinformatic searches of the proteomes of three diplomonads (*S. salmonicida, G. lamblia (syn. G. lamblia)*, and the related species *Trepomonas* sp. PC1), as well as two more distantly-related intron-rich relatives, *Kipferlia bialata*) and the oxymonad *Monocercomonoides* sp. PA203.

Among the 174 proteins for which we searched, species varied widely in the number of proteins found. As previously found for *Giardia* [13], all three diplomonads exhibited strongly reduced complements of spliceosomal proteomes, with 49 in *Trepomonas* sp. PC1, 44 in *S. salmonicida*, and 62 in *G. lamblia*. Notably, the three protein complements were highly overlapping: only 18 proteins were found in only a subset of the diplomonads, with most of the differences being proteins found only in *G. lamblia.* Some of these differences may be partially explained by incompleteness of the *Trepomonas* sp. transcriptome assembly used. Interestingly, the more intron-rich relatives exhibited larger complements (87 in *K. bialata* and 115 in *Monocercomonoides* sp*.*) (Additional file 9).

**Fig. 7:**
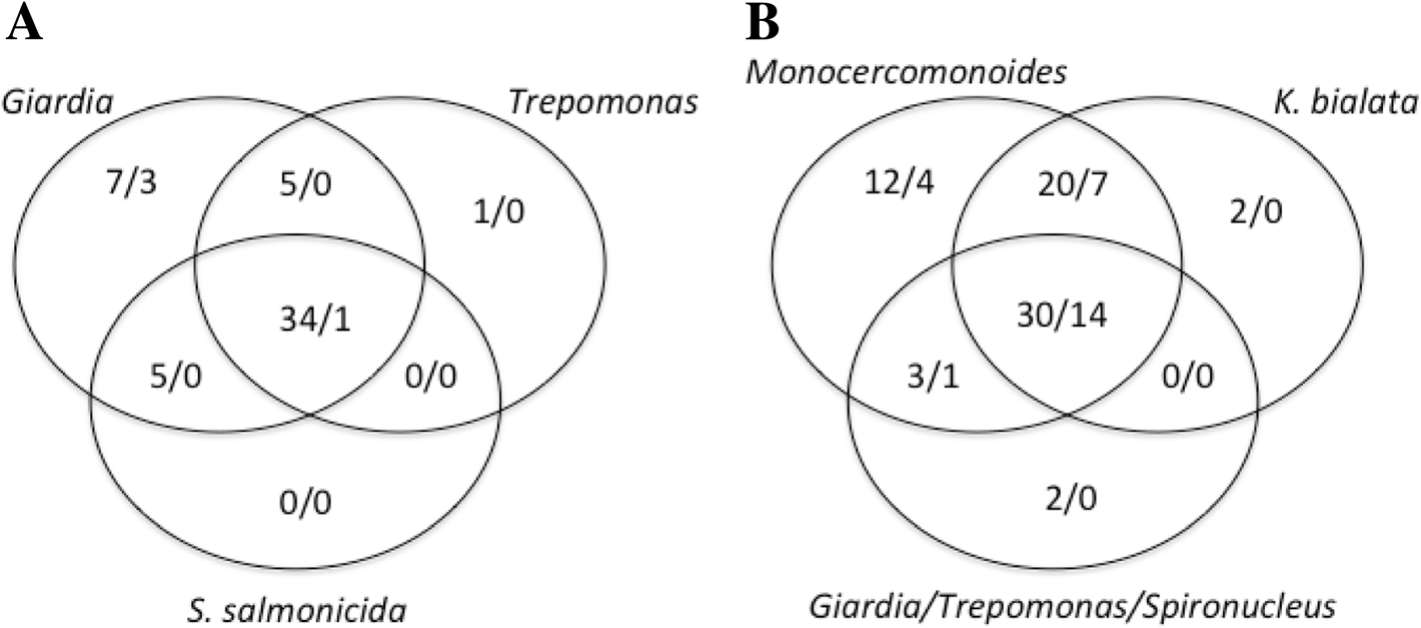
Comparison of spliceosomal protein retention patterns. Retention patterns of spliceosomal proteins compared in Venn diagrams. For (**a**)., proteins found in organism compared against X/Y where X = proteins found in both K (K = *K. bialata*) and M (M = *Monocercomonoides sp.* PA203), and Y = proteins found in either K or M. For (**b**)., proteins in organism (or group of organisms for G/T/S where G = *G. lamblia* S = *S. salmonicida,* T = *Trepomonas sp.* PC1.) compared against X/Y, but here X = proteins found in *H. sapiens, S. cerevisiae,* and *A. thaliana* and Y = proteins found in any combination of the two: *Hs* and *Sc*, *Hs* and *At,* or *Sc* and *At*

The specific proteins retained in the various metamonad organisms showed a strongly nested character – nearly all proteins retained in one or more diplomonad organism are retained in *K. bialata* and nearly all proteins retained in *K. bialata* are retained in *Monocercomonoides sp*. (Fig. 7). Further, the individual proteins that are found in all of our examined metamonads are twice more likely to be found in all three of our reference organisms. Interestingly, preliminary snRNA searches suggested a parallel difference in degree of transformation of the core spliceosome between these two organisms relative to the ancestral eukaryotic state – candidates for all five snRNAs were identified in *Monocercomonoides sp* but no candidates were identified in *K. bialata* (data not shown).

The Sm and Sm-like proteins (LSm) have been difficult to resolve in the past, and here, too, there is some ambiguity as to the exact identity of our set of LSM-domain containing proteins [38, 39]. The number of these Sm/LSm candidates, however, does mirror the overall spliceosomal protein-count trends, and *S. salmonicida* has only 4 of these proteins found in our search whereas *Monocercomonoides sp.* has 10 Sm/LSm candidates. Of note, the U1-associated proteins are nearly completely absent in all studied metamonads while, even with the reduced component numbers, Tri-snRNP and U5-associated proteins are the most well-represented groups (Table 1). Interestingly, the most pronounced difference is observed within the U2 snRNP, in which *G. lamblia* retains a core set of 8 U2-associated proteins while *S. salmonicida* lacks all U2-associated proteins and *Trepomonas* retains only one.

**Table 1:**
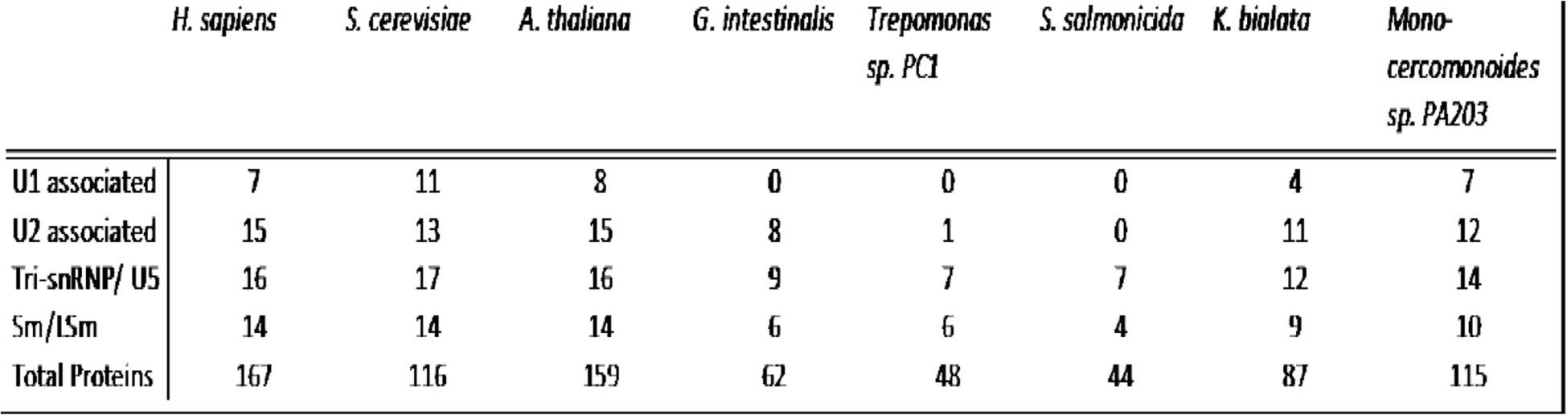
Summary of spliceosomal protein counts from selected subcomplexes. Spliceosomal proteins by subcomplex in this study. Select subcomplexes represented by organism and total number of associated proteins. Totals from *H. sapiens, S. cerevisiae,* and *A. thaliana* represent proteins shared between at least 2 of the 3 (see Methods)

## Discussion

### Intron conservation in diplomonad and parabasalid representatives

Identification of the first spliceosomal introns in *G. lamblia* and the parabasalid *T. vaginalis* revealed an unexpected level of intron structure and sequence conservation, with near-identical 5′ SS and fused BP + 3′ SS consensus sequences between the two species [15, 16] (Fig. 1b). Indeed, the *G. lamblia* ferredoxin intron was readily spliced from an expressed reporter gene construct in *T. vaginalis* (following 5′ SS ‘CT’ dinucleotide substitution to ‘GT’) [15] emphasizing the similarities in intron structure and splicing mechanism in these two organisms. While the *Spironucleus* introns display the fused BP + 3′ SS shared property, they also show some differences in splice site sequence preference and intron element spacing relative to *G. lamblia* or *T. vaginalis* introns, such as variation in 5′ and 3′ SS nucleotide sequences and typically an additional nucleotide insertion between the BP and 3′ SS sequences in the *Spironucleus* introns (Fig. 1b). Strict spacing distance between branch point sequence and 3′ SS is a feature seen in minor spliceosomal (U12-type) introns; however, the intron element consensus sequences of the *Spironucleus* introns and those identified in *G. lamblia* are closer matches to U2-type introns.

The structural properties of the identified *G. lamblia* introns suggest that intron elements may have particular importance in the spatial positioning of splice sites and the branch point ‘A’ during the splicing pathway, relative to other eukaryotes. These intron properties include an invariant distance between the branch point and 3′ SS, and extensive base pairing potential present not only between *trans*-spliced intron halves but also in the larger *cis*-spliced introns, such as the *Rpl7a* intron. We now provide evidence for intron structural potential in the longer *cis*-spliced introns in *Spironucleus spp.* and *T. vaginalis* which, similar to *G. lamblia*, may reduce the spatial distance between intron elements to lengths comparable to the shorter and uniformly-sized spliceosomal introns in these organisms. Interestingly, this includes the *S. vortens Rps15* 5′ UTR intron which appears to form a short stem-loop structure predicted to limit both the distance between 5′ and 3′ SS and the fused BP-3′ SS configuration (Fig. 1c). The conservation of extensive intron base pairing potential in a parabasalid (*T. vaginalis*) and diplomonads (*G. lamblia* and *Spironucleus spp.*) further indicates a shared requirement to maintain a specific spatial positioning of intron elements for efficient splicing and suggests that this property of spliceosomal introns may be much more phylogenetically wide spread than previously thought. It also further suggests possible intron evolution steps for the conversion of a *cis*-spliced intron to a *trans*-spliced intron [20]. Once internal base pairing potential is sufficiently long in a *cis*-spliced intron, a natural fragmentation position between the two complementary regions (i.e. within the loops of stem-loop structures) would allow the two halves of newly-created *trans*-spliced introns to associate efficiently, and the fragmentation event to be tolerated. Interestingly, the *Spironucleus* internal base pairing potentials of the introns identified thus far are relatively short compared to the ones observed in the *G. lamblia* introns (Figs. 1c and 3) and would likely be too short to allow efficient association if those introns were fragmented prior to significant extension of base pairing lengths. It would be interesting to know if there are any functional constraints on internal intron base pairing lengths in the *cis*-splicing mechanism in *Spironucleus* that explains why *trans*-spliced introns have not been detected in these species [23].

### *Spironucleus* snRNAs provide further insight into spliceosome structure evolution in diplomonads

Characterization of the U1, U2, U4 and U6 spliceosomal snRNAs from *G. lamblia* revealed that they are evolutionarily divergent and possess secondary structures and sequence motifs characteristic of both major (U2-dependent) and minor (U12-dependent) spliceosomal snRNAs [12, 32]. Given the relatively close evolutionary relationship of *Spironucleus* and *Giardia* we initially anticipated that the *G. lamblia* snRNAs would be most useful for the identification of the *Spironucleus* counterparts. Instead, fast apparent snRNA sequence and structural divergence within at least some diplomonads has occurred, making complete *S. salmonicida* and *S. vortens* snRNA complement identification particularly challenging. The *G. lamblia* snRNAs were only previously identified via their utilization of a conserved 3′ end ncRNA processing motif [32], that is not found to be conserved in these *Spironucleus* species.

The *Spironucleus* U2 snRNA candidates appear to be major/U2 snRNA-like, based on primary sequence comparisons with other representative U2 and U12 snRNAs (Additional file 10), but the noteworthy sequence divergence between the two species in the 3′ portions (~ 2/3 of the length) is further indicative of rapid snRNA evolution within these diplomonads. The *Spironucleus* U2 snRNAs appear to lack SL IV (Fig. 6b and c) and instead they are predicted to form an extended long SL III – a characteristic of minor U12 snRNAs [5, 8]. Similarly, the *G. lamblia* U2 snRNA [32] 3′ half may also fold into a similar conformation with a single long SL III. However, we find that neither the *G. lamblia* U2 snRNA [32] nor the *Spironucleus* U2 candidates contain the conserved SL III loop sequence ‘CUACUUU’ that is bound by the minor spliceosomal U12 snRNP-specific 65 kDa protein [40] and this protein was not detected in our spliceosomal protein analysis. Therefore, we conclude that the *Spironucleus* and *G. lamblia* U2 snRNAs are more likely bona fide U2-dependent/major spliceosomal components and are showing similar conserved 3′ structural features that may be indicative of U2 snRNA evolution in diplomonads.

The only other spliceosomal snRNA candidate we have been able to identify so far is the U5 snRNA in *S. salmonicida* (Fig. 6a). U5 snRNA is a shared component of both U2-dependent and U12-dependent spliceosomes and thus, its presence and structure is not particularly informative for explaining the major/minor duality of spliceosomal snRNAs in diplomonads. However, it is worth noting that our previous snRNA searches in *G. lamblia* did not reveal any U5 snRNA candidates with canonical features or that closely-resemble the predicted *S. salmonicida* U5. This seems to indicate that U5 snRNAs (assuming *G. lamblia* has a U5) may be quite divergent in both sequence and structure within the diplomonads. We predict that *S. vortens* also possesses a U5 snRNA despite our inability to identify a candidate and that this negative result may be the result of incompleteness of the *S. vortens* genomic data (Joint Genome Institute, unpublished data). However, given the amount of sequence divergence observed in the 3′ sections of the *Spironucleus* U2 snRNAs, it is also possible that the U5 RNAs are even more divergent between the two species increasing the difficulty in successfully identifying U5 in both.

Finally, some *Spironucleus* snRNAs, the U1, U4 and U6 snRNAs escaped detection in our searches, and therefore may also be evolutionarily-divergent compared to their *Giardia* counterparts. Although there are other possibilities to explain this, such as incomplete coverage during genomic sequence determination or inherent search strategy biases, it is reasonable to speculate that at least some of these “missing” snRNAs may be sufficiently divergent relative to *G. lamblia*, and to other eukaryotes in which co-variation models were developed, to escape ‘easy’ detection. Identification of the remaining snRNAs in *S. vortens* and *S. salmonicida* (and other diplomonads) should provide additional insight into the history of their respective spliceosomes and the unique paths of spliceosome evolution in different diplomonads.

### A high frequency of ancient spliceosomal introns in RP genes in diplomonads

Ancient spliceosomal introns are often maintained in intron-poor eukaryotes and particularly within RP genes. Consistent with this, we find that several (3 out of 6) of the confirmed *S. vortens* spliceosomal introns are ancient RP gene introns, with the *Rps4* and *Rps24* introns representing some of the most evolutionarily-conserved introns discovered to date. However, no single spliceosomal intron has been maintained in all of the diplomonad species studied thus far, indicating that spliceosomal intron loss is still ongoing and may eventually reach completion in members of this group.

### Diplomonads show a shared but markedly reduced set of spliceosomal proteins

The reduction in the number of spliceosomal proteins in *S. salmonicida* is not surprising given the observed phenomenon in other organisms. It is interesting to note, however, that many of these intron-poor organisms that shed spliceosomal proteins seem to be missing similar general sets of protein components. This could point to losses in a common ancestor or to parallel losses of the same proteins due to very different propensities to loss of different spliceosomal factors. Interestingly, the apparent dearth of U1 snRNP proteins echoes the complete loss of this snRNP in the highly-reduced spliceosomal system of *C. merolae*, as does the incomplete complement of ancestral LSm/Sm proteins. One notable caveat is that diplomonads appear to be generally quite transformed at the sequence level in general, and as such it is difficult to rule out the possibility that many of these proteins are in fact present, but are too divergent to be successfully identified via our current set of resources and bioinformatics tools. This is a particular challenge for spliceosomal proteins, since many harbor similar, common domains, which are hard to distinguish. Another possibility is that spliceosomal factors in highly-diverged organisms could take on secondary functions over the course of transformation, which could render ancestral factors redundant and thereby facilitate the observed component streamlining, as could the lessened need for efficiency that the reduced intron-density provides.

Another complicating possibility is the loss of domains from ancestral spliceosomal factors in transformed organisms. In this study we required retention of all conserved domains, an approach we favor in order to distinguish true spliceosomal factors from the myriad RRM-binding-domain containing proteins in eukaryotic genomes. However, this strategy has tradeoffs. For instance, diplomonad Prp8 (U5 200K) has apparently lost an N-terminal domain, which meant that it was initially excluded by our automated methods. While manual inspection allowed us to recover this well-studied, many-domain protein, other simpler or faster-evolving factors may have been overlooked by the conservatism of our method.

## Conclusions

Intron-poor eukaryotes are marked by constrained and extended intron splicing signals and reduced splicing machinery. In this study we find a remarkable level of sequence conservation of spliceosomal introns in *Spironucleus* species and evidence for additional structural constraints to position intron elements for efficient splicing – a feature apparently conserved in these diplomonad representatives and *T. vaginalis* (parabasalid) introns. The requirement for such positioning of intron elements is intriguing and points to a more simplified splicing mechanism(s) in these organisms. This coincides with changes in typically conserved spliceosomal structures including loss or modification of snRNA domains as observed in *G. lamblia* and *Spironucleus spp*. Such changes in snRNA structure may be concurrent with the loss of auxiliary spliceosomal proteins involved in splicing regulation and alternative splicing. Indeed, searches for spliceosomal proteins in *G. lamblia* and other eukaryotes revealed divergent homologs with several core spliceosomal components seemingly absent [12, 13, 19]. It will be interesting to determine whether snRNAs from other diplomonads and other divergent eukaryotes share these unusual features.

Finally, intron base pairing is proposed to mediate association of the known *G. lamblia trans*-introns and thus may be a required first step towards intron fragmentation and gene fission [20]. The conservation of intron base pairing in *cis*-spliced introns in diplomonads and a parabasalid and the large proportion of *trans*-spliced introns in *G. lamblia*, suggests that additional *trans*-spliced introns may await discovery in members of these groups.

## Methods

### Search for spliceosomal introns in *Spironucleus* species

Intron-poor eukaryotic genomes may have their introns concentrated within ribosomal protein-coding genes [16, 25], thus we reasoned that spliceosomal introns may possibly interrupt RP genes in *S. vortens*. Consequently, the complement of 80 ribosomal protein sequences from *Saccharomyces cerevisiae* was downloaded from the Ribosomal Protein Gene Database (http://ribosome.med.miyazaki-u.ac.jp/) [26] and each RP sequence was used as query in TBLASTN searches against the *S. vortens* expressed sequence tag (EST) library in the NCBI database and matching ESTs encoding RP sequences were obtained. In most cases, these searches unambiguously identified a matching *S. vortens* RP ortholog, however, 11 of the 80 *S. cerevisiae* RP protein sequences did not identify obvious RP gene orthologs. Next, the *S. vortens* RP EST sequences were used as queries in BLASTN searches against the *S. vortens* genomic sequences from the NCBI trace archive and for positive hits, 500 nt of additional upstream and downstream sequence was also downloaded. Genomic trace sequences were then aligned with corresponding ESTs manually and inspected for introns disrupting coding sequences. This strategy identified the *Rpl7a*, *Rpl30*, *Rps4* and *Rps24* RP gene introns.

In order to identify additional (and possible non-RP gene) introns, we utilized the pattern-matching software ‘Scan for Matches’ [27] in conjunction with the newly-identified *S. vortens* fused branch point and 3′ SS sequence consensus: 5′-RCTAACAARYTAG-3′ obtained from the identified RP gene introns. *S. vortens* raw genomic sequence reads were downloaded from the NCBI trace database (130 Mb genomic sequence) and was made into a concatenated file which served as the local database for our searches. Next, we searched the local database using 'Scan for Matches' and the pattern: 500 … RCTAACAARYTAG …500 (where ‘R’ and ‘Y’ represent a purine and a pyrimidine, respectively). We examined the hits for the presence of a potential 5′ SS in the regions upstream of the BP/3′ SS sequence. Next, sequences from the region downstream of the BP/3′SS from each unique hit were translated in the three possible reading frames using the ciliate genetic code (usual stop codons TAA and TAG codons are instead glutamine in *Spironucleus*) [28] and used as queries in BLASTP searches against the non-redundant protein sequence database at NCBI to determine if they encoded conserved protein-coding sequences. This strategy identified the *FolC-like* gene intron, the predicted hypothetical gene introns and the *Rps15* 5′ UTR intron.

In addition, we performed a genome-wide search for additional spliceosomal introns in *S. salmonicida*, taking advantage of available omics-level DNA and RNA-Seq data. The *S. salmonicida* genome sequence was downloaded from Ensembl release 41 (https://www.ebi.ac.uk/ena/data/view/GCA_000497125.1), and paired-end RNA-Seq was downloaded from NCBI (SRR948595) and aligned to the genome using HISAT2 2.1.0 [41] with the following non-default options: --pen-noncansplice 0, −-novel-splicesite-outfile. All genomic loci for which at least one read was annotated as spliced were retrieved from the genome using the splice sites file and custom Python/Perl scripts, and manually searched to identify likely intron sequences.

### 5′ rapid amplification of cDNA ends (RACE) and reverse-transcriptase polymerase chain reaction experiments

For 5′ RACE experiments, a total poly-adenylated RNA to cDNA library was first generated by reverse transcription, using *Spironucleus vortens* total cellular RNA and an oligo-dT reverse primer (oP-94) for first-strand cDNA synthesis (See Additional file 1 for primer sequences). Reverse transcriptase (RT) reactions were performed in 100 μL reactions containing: 1 μg of total *S. vortens* RNA, 1 X First Strand Buffer (Invitrogen), 10 μM DTT, 500 μM dNTPs, 200 pmol oP-94 reverse primer and 500 U SuperScript™ II RT (Invitrogen) to generate first strand cDNA, according to the manufacturer’s instructions and cDNA products were purified using E.Z.N.A.® Cycle Pure Kits. A control reaction without RT was performed as well to document successful removal of genomic DNA from total RNA samples. Purified cDNAs were then 3′ poly-dG tailed in 50 μL reactions consisting of 1X TdT Buffer (New England Biolabs, NEB), 250 μM CoCl_2_, 300 μM dGTP, and 10 U terminal deoxynucleotidyl transferase (NEB). Tailing reactions were incubated for 1 h at 37 °C, then heat inactivated for 10 mins at 70 °C. PCR reactions using either *Taq* polymerase (NEB) or Phusion polymerase (Thermo Scientific) were then performed on tailed cDNAs (and minus RT controls) using a poly-dC forward primer (oAR8), and gene-specific reverse primer (see Additional file 1 for primer sequences).

Reverse-transcription polymerase chain reaction (RT-PCR) was used to confirm expression of the predicted *S. vortens* U2 snRNA. RT reactions were performed as described above, except using 10 μg of *S. vortens* total RNA and oDM45 (Additional file 1) to generate cDNA, then treated with 5 U of RNase H (NEB) and incubated for 30 mins at 37 °C prior to heat inactivation for 20 mins at 65 °C. PCR was then performed on RNase H-treated cDNA samples using *Taq* polymerase (NEB) and U2-specific primers (Additional file 1). A –RT control sample was also generated including all steps but without addition of reverse transcriptase.

5′ RACE and RT-PCR amplified products were resolved on 3% agarose gels containing GelGreen (Biotium) nucleic acid stain and bands corresponding to predicted amplicon sizes were gel extracted and purified using E.Z.N.A.® Gel Purification Kits. Extracted bands were blunt-end cloned into the pJET1.2 vector, following the manufacturer’s instructions and DNA sequenced (Macrogen, USA) to confirm intron removal, mature 5′ ends sequences (RACE) and *S. vortens* U2 snRNA-candidate expression.

### Bioinformatic prediction of *Spironucleus* snRNAs

Spliceosomal small nuclear RNA coding regions were predicted in *S. vortens* and *S. salmonicida* genomic sequences using a combination of sequence motif and co-variation model (CM) search strategies that have been successfully employed for identifying snRNA gene sequences in many other eukaryotic genomic DNA databases [33]. Initially, optimized alignments of snRNA sequences from phylogenetically-diverse eukaryotes were downloaded from the Rfam database (http://rfam.xfam.org/) and used to generate CMs using the cmbuild tool from the Infernal software package [34]. Next, individual U-snRNA CMs were employed in cmsearch (Infernal software package) queries to identify snRNA-like sequences in *S. vortens* and *S. salmonicida* local DNA databases, with cmsearch *E* value cut-offs set to 10. In anticipation that *Spironucleus* snRNAs may be highly divergent (as observed for the *G. lamblia* snRNAs), all resulting cmsearch hits were examined manually for evolutionarily-conserved secondary structures or expected sequence motifs (e.g. BP interacting sequence for U2 snRNA). These searches successfully identified a U2 snRNA candidate in *S. vortens* and *S. salmonicida*.

U5 snRNA candidates were identified using 'Scan for Matches' queries specifying the conserved U5 snRNA loop I sequence ‘UGCCUUUUACY’ (allowing two mismatches) flanked by nucleotides capable of forming a 6 base pair helix (allowing G•U wobble pairs). For each hit, 100 nt of upstream and downstream sequence was then examined for the ability to form a longer stem-loop I consisting of conserved 1a/1b/1c helices and IL1 and IL2 internal loops, and the presence of a canonical Sm binding site (RAU_4-6_GR, where R is a purine). This strategy identified the *S. salmonicida* U5 snRNA candidate.

### Spliceosomal protein searches

To search for spliceosomal proteins, predicted proteomes were obtained from various species, either from NCBI (*Monocercomonoides sp. PA203*, assembly Mono14B; *Saccharomyces cerevisiae*, R64; *Homo sapiens*, GRCh38.p12), TAIR (*Arabidopsis thaliana*, Araport11), or from Goro Tanifuji (*K. bialata*). Spliceosomal proteins were collected from previous studies, and those that were present in at least two of *H. sapiens, S. cerevisiae,* and *A. thaliana* were compiled [42–44]. Initial spliceosomal protein candidates in the studied metamonads were identified by locally psiBLASTing queries against predicted protein data sets using BLAST version 2.7.1+ [45]. We used position-specific scoring matrices (PSSMs) that were generated using human spliceosome components as queries against the NCBI nr protein database. To avoid PSSM bias due to NCBI database overrepresentation of certain taxa, a restricted (R) PSSM was created by excluding plants (taxid:3193), animals (taxid:33208), Dikarya (taxid:451864), and Plasmodium (taxid:5820) from the search set. As some of the R PSSMs were limited to a few BLAST hits to align for the PSSM construction, non-restricted (NR) sets were also created by not excluding the above taxa in the initial, PSSM forming searches. psiBLASTs were run for 8 iterations with an E-value threshold of 10^− 6^.

To identify the conserved set of domains, known spliceosomal proteins were searched using an online HMMscan portal to identify annotated domains. The GenomeNet MOTIF tool (https://www.genome.jp/tools/motif/) was used to search Pfam and NCBI databases using default cut-off scores (set to an E-value of 1.0) for *H. sapiens* and *S. cerevisiae* domains, while information from Wang and Brendel (2004) was used to identify the domains in the spliceosomal proteins of *A. thaliana* [46]. Domains were considered conserved if they were present in all three of the *H. sapiens, A. thaliana,* and *S. cerevisiae* protein queries and domain lists were constructed for all spliceosomal proteins. For proteins not found in all three organisms, domains found in all the species containing the protein were used to construct the lists.

For metamonad proteins giving psiBLAST hits, raw HMMs of domains from above were downloaded from Pfam, and all psiBLAST hits were then searched with HMMsearch (HMMer 3.1b2) using default parameters with the associated domain lists [47]. Protein candidates were then removed if they did not contain all of the expected conserved domains. In some cases, proteins candidates were retained if they contained all but one of the expected conserved domains. The results of the LSM-containing proteins were screened for unique hits, and the total number of possible LSm/Sm proteins were counted from these hits. Finally, to remove false positive results, the screened psiBLAST hits were used as queries in a local BLASTp search against the human refseq_protein database. Proteins were considered “reciprocal” when one of the top 10 BLASTp hits matched the original PSSM-forming query and the matching protein was of similar size to the original query (± 50 aa for proteins under 300 aa and ± 20% for proteins larger than 300 aa).

### Intron secondary structure and RP gene intron conservation in eukaryotes

To identify possible conserved intron secondary structures, the collection of *S. vortens cis*-introns identified here and the annotated *T. vaginalis* introns (retrieved from TrichDB.org) were used as input for MFOLD [48] secondary structure predictions. MFOLD parameters were modified to force intron regions predicted to interact with spliceosomal machinery (i.e. 5′ SS, BP and 3′ SS) to be single-stranded and RNA folding temperatures were set to either 21 °C or 37 °C for *S. vortens* and *T. vaginalis* introns, respectively, based on the optimal growth temperatures for each organism. For each intron, the three most optimal (free-energy minimized) MFOLD secondary structural predictions were then examined for convincing secondary structure (extended helices) and the total single-stranded distance (excluding loop nucleotides) was determined.

To determine the phylogenetic conservation of *S. vortens Rps4* and *Rps24* introns in eukaryotes, orthologous *Rps4* and *Rps24* genes from representative eukaryotes were examined for intron insertion at the same relative position as *S. vortens* using the gene browser tool on the NCBI website (http://www.ncbi.nlm.nih.gov/gene/). Only introns found in the same phase and relative position of the RP gene-coding sequences were considered to be homologous introns. RP gene intron distribution was then mapped using a recent proposed eukaryotic tree from Burki (2014) [30].

## Additional files

Additional file 1: Table of primers used for 5′ RACE and RT-PCR experiments. This file contains the names and sequences for oligonucleotide primers used for RP cDNA synthesis and PCR in 5′ RACE and U2 snRNA RT-PCR experiments. (DOCX 15 kb)

Additional file 2: Spliceosomal introns in conserved protein coding genes from *Spironucleus vortens*. This file contains a table of intron-containing *S. vortens* protein coding sequences with corresponding genomic trace accession numbers and expressed sequence tags (ESTs) confirming intron splicing. (DOCX 17 kb)

Additional file 3: ClustalW2 alignment of ribosomal protein sequences. This file contains translated amino acid sequence alignments of intron-containing gene sequences from *S. vortens* with orthologs from various other eukaryotes. The alignments demonstrate *S. vortens* introns disrupt evolutionarily-conserved regions of RP gene sequences. (DOCX 32 kb)

Additional file 4: ClustalW2 alignment of *S. vortens* gene alleles containing intron sequences. This file contains nucleotide alignments for all intron-containing RP gene sequences found within *S. vortens* raw genomic sequence reads and readily identifies several unique RP gene alleles. (DOCX 20 kb)

Additional file 5: Base pairing potential in *S. salmonicida* introns. This file confirms secondary structural potential for the *S. salmonicida Rpl30* intron and an alignment showing the length distributions for the four known *S. salmonicida* introns (Xu et al. 2014). (DOCX 74 kb)

Additional file 6: Structural potential of *cis*-spliceosomal introns in *Trichomonas vaginalis*. This file contains a table showing the collection of known *T. vaginalis* spliceosomal introns and regions of introns predicted to form stem-loops by MFOLD software. Predicted single stranded distances between splice sites are also shown. (DOCX 18 kb)

Additional file 7: Clustal Omega alignments of 5′ RACE and RT-PCR sequencing products with predicted mRNA sequences lacking introns and U2 snRNA. This file contains nucleotide alignments of our 5′ RACE products and U2 snRNA RT-PCR sequencing results with the predicted sequence of RP mRNAs following removal of the proposed intron, and the proposed U2 candidate. (DOCX 17 kb)

Additional file 8: Evolutionary conservation of *Rps4* and *Rps24* gene introns in eukaryotes. This file contains a table of all organisms examined for *rpS4* and *rpS24* gene introns. When possible the length and sequences for each identified intron are provided. (DOCX 31 kb)

Additional file 9: Table of spliceosomal proteins found in our searches (see Methods). Numbers in red indicate that the initial psiBLAST searches had 0 hits, and HMM searches were not a part of their disqualification. Numbers in grey indicate that the protein was missing a domain from the conserved set. Sm and LSm proteins are grouped together. Hs - *H. sapiens,* Sc - *S. cerevisiae*, At - *A. thaliana*, Gi - *G. lamblia,* T - *Trepomonas sp.* PC1, Ss - *S. salmonicida*, Kb - *Kipferlia bialata sp.*, M - *Monocercomonoides sp.* PA203. (PDF 57 kb)

Additional file 10: Primary sequence comparison of *Spironucleus* U2 snRNA candidates with U2 and U12 snRNAs from representative eukaryotes. This file contains U2 and U12 snRNA nucleotide alignments showing *Spironucleus* snRNA regions which are representative of either major (U2) or minor (U12) snRNA class. (DOCX 24 kb)

## Data Availability

Additional information is provided in the Supplemental Figures.

## Abbreviations

BP: Branch point
cDNA: Complementary deoxyribonucleic acid
CM: Co-variation model
EST: Expressed sequence tag
IL: Internal loop
LECA: Last eukaryotic common ancestor
mRNA: Messenger ribonucleic acid
nt: Nucleotide
ORF: Open reading frame
PCR: Polymerase chain reaction
RACE: Rapid amplification of cDNA ends
RP: Ribosomal protein
RT: Reverse transcription
snRNA: Small nuclear ribonucleic acid
SS: Splice site
UTR: Untranslated region
