# Supplementary material for: Patterns of conservation of spliceosomal intron structures and spliceosome divergence in representatives of the diplomonad and parabasalid lineages
Source: BMC Evol Biol. 2019 Aug 2;19:162. doi: 10.1186/s12862-019-1488-y (PMC6679479; doi:10.1186/s12862-019-1488-y)
Supplement: Supplementary file 1 — Table of primers used for 5′ RACE and RT-PCR experiments. This file contains the names and sequences for oligonucleotide primers used for RP cDNA synthesis and PCR in 5′ RACE and U2 snRNA RT-PCR experiments. (DOCX 15 kb) [file 12862_2019_1488_MOESM1_ESM.docx]

|  |  |  |  |  |
| --- | --- | --- | --- | --- |
|  | **Primer name** | **Experimental use of the primer** | **Primer sequence (5′ - 3′)** |  |
|  | oDM30 | Rpl7a reverse primer for 5′ RACE | ATGGCGTGGACGTAGTCGACC |  |
|  | oDM32 | Rpl30 Reverse primer for 5′ RACE | GAGCTTGGCCTTCAGGTTGCG |  |
|  | oDM33 | Rps4 reverse primer for 5′ RACE | CATGAAGCCGACGGAGAACTGG |  |
|  | oDM35 | Rps24 reverse primer for 5′ RACE | TTACCACCGTGCTTGGTGTGGC |  |
|  | oDM37 | Rps15 reverse primer for 5′ RACE | GACGGCAGCCTTGTTGAGG |  |
|  | oDM44 | U2 forward primer for RT-PCR | CAAGTTTCGGCCCTGGTAAAGC |  |
|  | oDM45 | U2 reverse primer for RT-PCR | CGAGGGAGTGGCCCAGACCG |  |
|  | oP-94 | Poly-dT primer for 5′ RACE | AATAAAGCGGCCGCGGATCCAATTTTTTTTTTTTTTTTT(A/C/G) |  |
|  | oAR8 | Poly-dC primer for 5′ RACE | CTCCCGCTTCCAGATCTCGAGCCCCCCCCCCCCCCC(G/A/T) |  |
|  |  |  |  |  |

**Additional file 1 – Table of primers used for 5′ RACE and RT-PCR experiments.** List of primers used in 5′ RACE and RT-PCR experiments for ribosomal protein mRNA and U2 snRNA candidate analysis.
