# Supplementary material for: Patterns of conservation of spliceosomal intron structures and spliceosome divergence in representatives of the diplomonad and parabasalid lineages
Source: BMC Evol Biol. 2019 Aug 2;19:162. doi: 10.1186/s12862-019-1488-y (PMC6679479; doi:10.1186/s12862-019-1488-y)
Supplement: Supplementary file 2 — Spliceosomal introns in conserved protein coding genes from Spironucleus vortens. This file contains a table of intron-containing S. vortens protein coding sequences with corresponding genomic trace accession numbers and expressed sequence tags (ESTs) confirming intron splicing. (DOCX 17 kb) [file 12862_2019_1488_MOESM2_ESM.docx]

**Additional File 2 - Spliceosomal introns in conserved protein coding genes from *Spironucleus vortens*.**

Genomic sequences encoding predicted and EST-confirmed spliceosomal intron-containing genes from *S. vortens* are shown with intron sequences in lower case red text and start/stop codons are bolded. GenBank expressed sequence tags (ESTs) confirming intron splicing and the phase of intron insertions are indicated.

| **Host Gene** | **Gene Coding Sequence 5’-3’**  **(introns in red text)** | **Genomic trace**  **accession** | **Intron Phase** | **Confirming ESTs** |
| --- | --- | --- | --- | --- |
| *Rpl7a* | **ATG**CACCGCTGCCAAGCCGAACACGTCAACAACCTGACCCGCATGGTCAAGTGGCCCGCCTACATCCGCATCCAGCGCCAGAAGGCCCTCCTCCAGCACCGCCTGAAGGTCCCCGGCGTCGTCAACATGTTCCGCAACCCGCTGAACGCCAACGCCACCAAGGAGATCCTGAAGTTCGCCGCCAAGTACCAGCCGGAGACCAAGGAGGCCAGACAGCAGCGCCTTGTCCAGGCTGCCGACAAGAAGACCACCATCAACGCCCCAGTGTCCTTCAACTACAACATCCACAAGGTTGTTGAGGCCGTCGAGAAGAAGGAGGCCAAGCTGGTCCTCATCGCCCACGACGTCGACCCAATCGAGgtaagtctaaactaactgtgatccgcggatcgcttgagttacgaaactttgctaacaaactagCTCGTCCTGTACCTGCCAACCCTCTGCCACAAGAACAACATCCCATATGCCATCGTTCGCTCCCGCACCGAGCTCGGCAAGCTGGTTCACTGCACCAAGTGCACCTCCATCGCCTTCACCACCATCAAGCCGGAGGACACCGCCGCCTTCAAGTCCATCCTGGACACTGTTGCCCACGAGGTCGACTACGTCCACGCCATCAAGACCCACGGTGGTGTTTCCCGCTCCAACAAGTCCCTTGCTAAGGAGGCCAAGAAGAACAAGATCGGCAAGAAA**TGA** | Ti:2141515448:16-733 | 0 | GH187119.1,  GH187120.1,  GH184167.1,  GH184166.1,  GH195615.1 |
| *Rpl30* | **ATG**GATCGCGTATCTgtgagttgaaacaaactgagaccagaaacctggatccagtacaaacgaaactttactaacaaactagAAGAAGTCTTCTGAATCGGCCGCCTTGCAGCTTGCTCTTGTCGTCAAGTCCGGCAAGTACACCCTTGGTGTCAACCAGGCTCTTAAGTCCATCCGCAACCTGAAGGCCAAGCTCGTCATCATCACCTCCAACCTTCCACCCCTGGTCGCCTCCCAGATCGAGTACCTCTGCATGCTCAGCGGCATCCCAGTCCACGCCTTCCCGTCCAACTCCCGCGAGTTCGGCGTTACCCTCGGTAAGCAGTTCAACGTCGGCGTTATGGCCGTGACTGAAGCCGGCGACGCTGACCTCGCTGCCTTCAAG**TGA** | Ti:2141591039:516-129 | 0 | GH194962.1,  GH190822.1,  GH188072.1,  GH188071.1 |
| *Rps4* | **atg**gtaagtctaaaatgtgtgcgcacggcgcatcatctatttgcttgaactaacaaactaggctcgtggtccaaaacttcatatgaaacgtcttaacgctccatcccactggcagcaggacaagcttggcggcatctactccaccaagtgcaacctctccacccacaggatcaatgagtgcgtcccaatgtccctcgttctccgcaaccgcctgaacctcgccaagaccctccgcgagtgcaagctcatcctcgacaccaagaacatcctggttgacggcaaggtccgcaccgactcccagttctccgtcggcttcatggacgtcctcgaggtcaagaagctcaacaagctgtaccgcatcctcttcgacatcaagggccgcctgaccctccagccgatcgacaagaaggaggccgagttcaagctcctccgcatcaacaaggtcttcctcggtgagaagggcgtccgctacggtgtctctcacgacggccgcaccatccgcttcctcccggacgacgtcaaggtcaacgacaccgtcaagttcgacctgaagaccggcaacatcgttgagaaggcccagttcaacatcggccagatggcctgtgtcaccatcggtcagaacgtcggctccatcggcaagatcacccaggtcgacccgcacaacggctcctacaccatggtccacctcgtcgacgccgccggccagaagttcatcacccgccgcgagaacgtcttcatcctcggcaaccagggtaactccttcatctctatccccaaggagaagggcgtcaagccgaccatcttccaggagcgtgacctccgcctggcctccatcgccaagcaccagagaaacgag**tga** | Ti:2141550682:123-914 and  Ti:2141654036:986-256 | 0 | GH185743.1 |
| *Rps12* | **ATG**TCAACgtaagtctagagctgagcagtcaactttactaacaaaatagTGACCAACTGAAGACTTTCTGTAAGAAGATCCGCGTCCACGGTGCTATGGTCTCCGGCGTCCGCCAGGTCGTGCGCGCCGTCGAGAACCACGCCACCTCCAACGTGAAAGTCATTCTCCTGGCCAACGACTGCAAGGAAGCCGGCATCAAGAACCTCGTCAAGGCCCTCGCCAAGCAGCACTCCATCGGCGTCTGCGAGAAGTTCGGCGCCGCCCACCTCGGCGAGCTCGCCCACCAGTACGTGATCAAGGGCCACGTCACCGAGGGCAAGATCGGCAAGGTCAGAAACGCCTCCTGCATGGCCATCCAGAACTTCGGCACCCTCACCGCTGAGGATCAGGCCGCTTTCAACGCTCTCCTCCAG**TGA** | Ti:2141614707:43-458 | +2 | GH185220.1 |
| *Rps24* | **ATG**CAGATCAAGTATCGCGAAATTGTCAACAACCCGATCCTCGATCGTACTCAAATGgtaagtctaaatctcatgtataactaatactaacaagttagAAGCTCAAGATCGTCCACCCAGGTAAGTCCGTGGGTACCATCGAGGCTCTCCGCGAGCTCGTCCAGAAGGATCGTAAGATCAAGGACATCAAGCAGGTTGTCGTCTTTGACTGCCACACCAAGCACGGTGGTAACCTCAGCACTGCTTCTTGCCACATCTACGGCAACGTTGAGACCCTGAAGAAGGTTGAGCCGAAGTACACCATCATCCGCCTTGGTTACATCGAGAAGCCGAAGCCAGTCTCCCGCAAGATGATCAAGAACCACAAGAACAAGCTCATCCGCAAGTTCGGTACTGCCAAGAGCAAGATCGTCATGTCTGGTAAGAAGAAC**TGA** | Ti:2141541737:116-549 | 0 | GH184038.1,  GH193644.1,  GH184039.1,  GH192698.1,  GH192697.1 |
| *FolC-like* | **ATG**TAGTACCCTCAAGTGCTTgtaagtcaacttttgccatcaaacttttgctaacaaattagGATTCCCTCTCAAAAGTATCATAAACTGTCAAGTCGCATTGGTCATAGCTACCAGATCTACTAAATAAGTAATTTCGACCGGAATTGCTGTTTCACGTCACTGGTTCAAAAGGTAAAACGTCTATTTCGCACTATTTGAGCAAAAATATAACAAATAGCGGACTGTTTACATCTCCCCACCTTATTAGCTTCCGCGATAGAATCAAGGTGAACAACACGCCTCTTACACCACTAGAGTTTACAACCCTATATAATTATCACTTATCCAATATATAGAATTTACCACCGTTTCAAAAATCGTTCGTGTTGGCTCAAAACCACTTCTCAAATCTGTCGCTCCCCGTTCAGATTTTTGAGGTCGGTATAGGCGGCTTGCACGACTCCACATAAGCA**TGA** | Ti:2141479887:480-23 | 0 | GH189627.1 |
| *Rps15*  (Predicted 5’ UTR intron­) | TTCATTTTGATATTAAGTCTTTTTAGTTATGTTTTCCACACCTTTTCTTTTGGGTAACTAATTgtaagtctaaatctattgcgaaactttgctaacaaggtcctggaacgggccctagA**ATG**GGTCGTACTAATGTTCTCAATGACGTTCTCAAGCAGATCACCAACGCTCAGCGCCTTGGCAGACGCCAGTGCATCCTGCACCCAGTCAACTCCGTCACCCTGAAGGTCCTGGAGATCATGCAGAAGGAGGGCTACATCGGCGACTTCACCTTCGTTGATGACCGCCGCGGCAACAAGGTCGTCGCGAACCTGACCGGCCGCCTCAACAAGGCTGCCGTCATCTCCCCGCGTTTCGACGTCTCCCACAACGACCTCTCCAAGTGGGTTGTCAACCTCCTCCCGTCCCGCCTCTTCGGCCACATCCTGCTCTCCACCACCGTCGGCATCATCGACCACAACGAGGCCCAGCACCGCAACATTGGAGGCAAGATAATCGGTTTCTTCTAC**TGA** | Ti:2141512834:214-725 |  | None |
| Hypothetical ORF 1  (Predicted – 3’ end unknown­) | **atg**tccgaaacctcgtcctccagcgacgctggagacgctttcgagtaatatcgtaggtctaattgatatggataactttactaacaaactagTGCAATAGCGCCAGAAAAAGAACCAAGAAATCGAAGAATTAAAGAATCAAACGCAGTAACTGCTCTTGTAAACCCAGGAAAAGGAATAAATCGACCCGTACGCAGATATTATGAACTCCCTAAAAGACACGAAAAATCTCGTCGGCAAAGACGTCGACTAATGGGAGCAAACGATTAAAACTGACTTCGCTGAGCCAGATGTTCAAGATCAGAATGACATCTTATAAGAGACACAACAATCACTCGCTCAACAATCACAACTCATTGATGTCGCGTCGCCAGTTCATGAATAGCAGTCTCTGGCTGCCACCATGTCGCCGCAGTAGCCGCAACCTGTCGACCTGACAACATAAAATGACACATTGCGAATGGAAAAAGAGGACTTCTATTTTAAAAAATCGCTGAGCCAACAGTAGAAACTGCAGGCTGTATTGAACTAGCAAAACAAATAGTAAAAGCTGATAAAGTAGAAAAACAAAGACGAATTGGAGTAAATAAGCTAGCTCTTCAAAACTATGGTAAATAAACCAAATCCATAGGCGGCCAACTAGCAAGTAAAGCATGCCCTCGTCGCGCCCCAGCAGCAAATCTAAAACTACTTCGCCGACTACCAGAATTTCATGCTCAGCGTCCAGTCGATATCGCCCATAATCATCCAGAGGGCCGCTA | Ti:2141664662:761-1 | +1 | None |
| Hypothetical ORF 2  (Predicted) | **ATG**GAAGCAAGTCACTATGAATAGCTTAGCTgtaagtctaaattcgatcgtataaactctgctaacaaaatagAACTCGAGGCCCTGACGAAGATTTTGAAGCTCAATTTGACTCGTGAGCAGCTATCTGCATTGATGGAGTTGACCGAGACGGGCGTGAATCCGGAGGCAATTGCGGCCACAATCGCGGAGATCATG**TGA** | Ti:2141558755:563-763 | +1 | None |
| Hypothetical ORF 3  (Predicted) | **ATG**TCTGATAAATTACCATTTGAGGTTCTTTCAGACCAGGAAAAGTAGTAAGATGAAATgtaagtctaataaatacaggaaactttcgctaacaagatagAATTAAGAAGCTTACGTATATTGCTTCATAGCAGCAACATATGATCTAACTGTCTGAATTGACATTTAAATAGTTGGCTAGTTCATTTGATGCAAGCTCAAAAATGCTTCAATAATATGTTACCGATCTTCATGAGGCAGTCAACATAACTCAGAAATTCAAAAAA**TGA** | Ti:2141517615:182-450 | +2 | None |
