# Supplementary material for: Patterns of conservation of spliceosomal intron structures and spliceosome divergence in representatives of the diplomonad and parabasalid lineages
Source: BMC Evol Biol. 2019 Aug 2;19:162. doi: 10.1186/s12862-019-1488-y (PMC6679479; doi:10.1186/s12862-019-1488-y)
Supplement: Supplementary file 3 — ClustalW2 alignment of ribosomal protein sequences. This file contains translated amino acid sequence alignments of intron-containing gene sequences from S. vortens with orthologs from various other eukaryotes. The alignments demonstrate S. vortens introns disrupt evolutionarily-conserved regions of RP gene sequences. (DOCX 32 kb) [file 12862_2019_1488_MOESM3_ESM.docx]

**Additional File 3 - ClustalW2 alignment of ribosomal protein sequences.**

Translated nucleotide sequences for *S. vortens* intron-containing RP genes are aligned with RP sequences from various eukaryotes using ClustalW2. RP proteins are represented in single letter amino acid code with an asterisk (‘*’) indicating an in-frame stop codon. Amino acids which are conserved in four or more organisms are highlighted in black and translated intron sequences are in grey highlighting. Sequences used for alignments are: **(A) RP L7a** – *Homo sapiens* (Hs – NCBI Accession: EAW81017), *Arabidopsis thaliana* (At - NP_191846), *Trypanosoma brucei* (Tb - XP_846969), *Giardia lamblia* (Gl - XP_001706321) and *Spironucleus vortens* (Sv); **(B) RP L30** – *Homo sapiens* (Hs - NP_000980), *Drosophila melanogaster* (Dm - NP_524687), *Trypanosoma cruzi* (Tc - XP_810701), *Saccharomyces cerevisiae* (Sc - NP_011485), *Trichomonas vaginalis* (Tv - XP_001584528) and *Spironucleus vortens* (Sv); **(C) RP S4** - *Homo sapiens* (Hs - EAW71816), *Drosophila melanogaster* (Dm - NP_729871), *Arabidopsis thaliana* (At - NP_001189539), *Saccharomyces cerevisiae* (Sc - NP_012073), *Dictyostelium discoideum* (Dd - XP_644913) and *Spironucleus vortens* (Sv); **(D) RP S12** – *Homo sapiens* (Hs - NP_001007), *Saccharomyces cerevisiae* (Sc - NP_015014), *Dictyostelium discoideum* (Dd - XP_638666), *Arabidopsis thaliana* (At - AAD15398), *Trypanosoma brucei* (Tb - XP_828505), *Encephalitozoon cuniculi* (Ec – CAD24962); *Spironucleus vortens* (Sv) **(E) RP S24** - *Homo sapiens* (Hs - EAW54618), *Drosophila melanogaster* (Dm - NP_611693), *Saccharomyces cerevisiae* (Sc - NP_012195), *Arabidopsis thaliana* (At - NP_187143), *Perkinsus marinus* (Pm - XP_002766228), *Tetrahymena thermophila* (Tt - XP_001025062), *Thalassiosira pseudonana* (Tp - XP_002291077) *Giardia lamblia* (Gl - XP_001709806), *Spironucleus vortens* (Sv).

**(A) Ribosomal protein L7a (*Rpl7a*)**

Hs --------MPKGKKAKGKKVAPAPAVVKKQEAKKVVNPLFEKRPKNFGIGQDIQPKRDLT 52

At -------MAPK----KGVKVAS------KKKPEKVTNPLFERRPKQFGIGGALPPKKDLS 43

Tb MAGKEVKKAVKPTKKAGVPYKKPEVTQKKAKASAAAPSPFVARPKDFGIGRDVPYARDLS 60

Gl -------------------------------MSKVSGSDIKRALAVPENKSRSKCDFDLT 29

Sv ---------------------------------------MHRCQAEHVN--------NLT 13

Sv (with intron)---------------------------------------MHRCQAEHVN--------NLT 13

Hs RFVKWPRYIRLQRQRAILYKRLKVPPAINQFTQALDRQTATQLLKLAHKYRPETKQEKKQ 112

At RYIKWPKSIRLQRQKRILKQRLKVPPALNQFTKTLDKNLATSLFKILLKYRPEDKAAKKE 103

Tb RFMRWPTFVTMQRKKRVLQRRLKVPPALNQFTKVLDRSSRNELLKLVKKYAPETRKARRD 120

Gl PFVRWPRQVRIQRQKAVLQRRLKVPPTVNQFMNPISRNLTNEIFNLARKYSPESKEEHKA 89

Sv RMVKWPAYIRIQRQKALLQHRLKVPGVVNMFRNPLNANATKEILKFAAKYQPETKEARQQ 73

Sv (with intron)RMVKWPAYIRIQRQKALLQHRLKVPGVVNMFRNPLNANATKEILKFAAKYQPETKEARQQ 73

Hs RLLARA-EKKAAGKGDVPTKRPPVLRAGVNTVTTLVENKKAQLVVIAHDVDPIELVVFLP 171

At RLLNKA-QAEAEGK-PAESKKPIVVKYGLNHVTYLIEQNKAQLVVIAHDVDPIELVVWLP 161

Tb RLTKVAEEKKKNPKGTVSTKAPLCVVSGLQEVTRTIEKKTARLVLIANNVDPIELVLWMP 180

Gl RLLQIA-DAKANGKPLPEKSNKLVIASGIRRITSLVESKRAKLVLIANDVDPLELVLWLP 148

Sv RLVQAA-DKKTTIN-APVSFN-----YNIHKVVEAVEKKEAKLVLIAHDVDPIELVLYLP 126

Sv (with intron)RLVQAA-DKKTTIN-APVSFN-----YNIHKVVEAVEKKEAKLVLIAHDVDPIEVSLNQL 126

Hs ALCRKMGVPYCIIKGKARLGRLVHRKTCTTVAFTQVNSEDKGALAKLVEAIRTNYNDRYD 231

At ALCRKMEVPYCIVKGKSRLGAVVHQKTAAALCLTTVKNEDKLEFSKILEAIKANFNDKYE 221

Tb TLCRANKIPYAIVKDKARLGDAIGRKTATCVAFTDVNAEDQAALKNLTRSVNARFLARSD 240

Gl TLCHKMGVPYAIVRTKGDLGKLVHLKKTTSVCFTDVNPEDKPTFDKILAAVAH--EVDYA 206

Sv TLCHKNNIPYAIVRSRTELGKLVHCTKCTSIAFTTIKPEDTAAFKSILDTVAH--EVDYV 184

Sv (with intron)*

Hs EIRRHWGGNVLGPKSVARIAKL-EKAKAKELATKLG 266

At EYRKKWGGGIMGSKSQAKTKAK-ERVIAKEAAQRMN 256

Tb VIRRQWGGLQLSLRSRAELRKKRARTAGNDAAAKAA 276

Gl KAMKTYGGGVRRED----EAQ--------------- 223

Sv HAIKTHGGVSRSNKSLAKEAKKNKIGKK-------- 212

**(B) Ribosomal protein L30 (*Rpl30*)**

Hs MVAAKKTKKSLESINSRLQLVMKSGKYVLGYKQTLKMIRQGKAKLVILANNCPALRKSEI 60

Dm MVAVKKQKKALESTNARLALVMKSGKYCLGYKQTLKTLRQGKAKLVLIASNTPALRKSEI 60

Tc --MAKKNKTKVDTINTKIQLVMKSGKYVLGTKQTLKTLRQGRSKLVVISSNCPPIRKAEI 58

Sc MAPVKS----QESINQKLALVIKSGKYTLGYKSTVKSLRQGKSKLIIIAANTPVLRKSEL 56

Tv -MGRKKLSRARESIINSLSLVTKSGKYSLGISQTLKSLRNGEAKLVIFASNVAPADRSLI 59

Sv MDRVSK--KSSESAALQLALVVKSGKYTLGVNQALKSIRNLKAKLVIITSNLPPLVASQI 58

Sv (with intron)MDRVSVS*

Hs EYYAMLAKTGVHHYSGNNIELGTACGKYYRVCTLAIIDPGDSDIIRSMPEQTGEK 115

Dm EYYAMLAKTEVQHYSGTNIELGTACGKYFRVCTLSITDPGDSDIIRSLETA---- 111

Tc EYYCTLSKTPMHHYAGNNLDLGTACGRHFRSCVLSITDVGDSDITSA-------- 105

Sc EYYAMLSKTKVYYFQGGNNELGTAVGKLFRVGVVSILEAGDSDILTTLA------ 105

Tv EYYAMLSGCDILPFDGDNVDLGTACGKYFRSSVISIIDAGESEILKMIKQKDE-- 112

Sv EYLCMLSGIPVHAFPSNSREFGVTLGKQFNVGVMAVTEAGDADLAAF-------- 105

**(C) Ribosomal protein S4 (*Rps4*)**

Dm MARGPKKHLKRLAAPKAWMLDKLGGVFAPRPSTGPHKLRESLPLLIFLRNRLKYALNGAE 60

Sc MARGPKKHLKRLAAPHHWLLDKLSGCYAPRPSAGPHKLRESLPLIVFLRNRLKYALNGRE 60

Hs MARGPKKHLKRVAAPKHWMLDKLTGVFAPRPSTGPHKLRECLPLIIFLRNRLKYALTGDE 60

Dd MARGPKKHLKRLAAPNHWMLDKLSGKWAPRPSSGPHKLRECLPLILVLRNRLKYALTKKE 60

At MARGLKKHLKRLNAPKHWMLDKLGGAFAPKPSSGPHKSRECLPLVLIIRNRLKYALTYRE 60

Sv MARGPRKHLKRLNAPKHWLLDKMGGIWAPRPTNGPHGLRECIPLILILRNRLHYANTYAE 60

Sv (with intron)MVSLKCVRTAHHLFA*

Dm VTKIVMQRLVKVDGKVRTDPTYPAGYMDVITLEKTGEFFRLVYDVKGRFVIHRISAEEAK 120

Sc VKAILMQRHVKVDGKVRTDTTYPAGFMDVITLDATNENFRLVYDVKGRFAVHRITDEEAS 120

Hs VKKICMQRFIKIDGKVRTDITYPAGFMDVISIDKTGENFRLIYDTKGRFAVHRITPEEAK 120

Dd VTLILMQRLVKVDGKVRTDPNYPAGFMDVISIEKTKENFRLLFDPKGRFTLQRITPEEAK 120

At VISILMQRHIQVDGKVRTDKTYPAGFMDVVSIPKTNENFRLLYDTKGRFRLHSIKDEEAK 120

Sv TNMILKDKNVLIDNKPRIDPTFPIGFMDVFEIPKVHKVFRVLYDVKGRFTLIPIQSNEAG 120

Dm YKLCKVKKTQLGAKGVPFLVTHDGRTIRYPDPLIHANDSVQVDIASGKITDYIKFDSGNL 180

Sc YKLGKVKKVQLGKKGVPYVVTHDGRTIRYPDPNIKVNDTVKIDLASGKITDFIKFDAGKL 180

Hs YKLCKVRKIFVGTKGIPHLVTHDARTIRYPDPLIKVNDTIQIDLETGKITDFIKFDTGNL 180

Dd FKLARVTRVETGNQGIPYVHTDDGRTIRYPDPAISIHDTIKIDIESGKITAFIPFEVNNL 180

At FKLCKVRSIQFGQKGIPYLNTYDGRTIRYPDPLIKPNDTIKLDLEENKIVEFIKFDVGNV 180

Sv FKLCRVQKIFLGDKGMPYLVTHDARTIRFPHPDIKTNDTIKINLKTGKIDEWYKFDIGKV 180

Dm CMITGGRNLGRVGTVVNRERHPGSFDIVHIKDSQGHVFATRLTNVFIIGKGNKPYISLPK 240

Sc VYVTGGRNLGRIGTIVHKERHDGGFDLVHIKDSLDNTFVTRLNNVFVIGEQGKPYISLPK 240

Hs CMVTGGANLGRIGVITNRERHPGSFDVVHVKDANGNSFATRLSNIFVIGKGNKPWISLPR 240

Dd CMIVGGHNLGRVGAVTHREKHPGSFDIVHVTDTAGHQFATRLSNVFIIGKASQTFVSLPA 240

At VMVTGGRNRGRVGVIKNREKHKGSFETIHIQDSTGHEFATRLGNVYTIGKGTKPWVSLPK 240

Sv VMVTGGRNCGRIGTIQAIDKHMGSYTMIRMKDSEGAEFITRLCNVFIIGNDS-PAVTVPS 239

Dm GKGVKLSIAEERDKRLAAKTH------ 261

Sc GKGIKLSIAEERDRRRAQQGL------ 261

Hs GKGIRLTIAEERDKRLAAKQSSG---- 263

Dd GKGVRRSRVDERNAALKRRGEKIETVA 267

At GKGIKLTIIEEARKRLSAQQA------ 261

Sv TKGIRPDIIKNRELRLRSIAK------ 260

**(D) Ribosomal protein S12 (*Rps12*)**

Hs ----------MAEEGIAAG-GVMDVN----TALQEVLKTALIHDGLARGIREAAKALD-- 43

Sc MS-DVEEVVEVQEETVVEQTAEVTIE----DALKVVLRTALVHDGLARGLRESTKALT-- 53

Dd --------MEGDAPVIAANPLEKNTDP--MVALQKVIKESLAVQGVARGLHETVKALD-- 48

At MSGDEAVAAPVVPPVAEAAVIPEDMDV--STALELTVRKSRAYGGVVRGLHESAKLIE-- 56

Tb MAEETSLVADKVPEPAVIDAVADAMPDSLEDALRIVLMKARETNGLICGLSEVTRALD-- 58

Ec ------------MSEMQEPMMEPEMTL--QEALSKVCKVSRTYCKLSRGAKETTKKML-- 44

Sv ------------------------MST---DQLKTFCKKIRVHGAMVSGVRQVVRAVENH 33

Sv (with intron)------------------------MST---QVQS*

Hs -KRQAHLCVLASNCDEPMYVKLVEALC--AEHQINLIKVDDNKKLGEWVGLCKID---RE 97

Sc -RGEALLVVLVSSVTEANIIKLVEGLANDPENKVPLIKVADAKQLGEWAGLGKID---RE 109

Dd -KRTARLCVLASNCDEPNFVRLVKALA--TEHNIPLIEVPDNKALGEWAGLCKLD---KD 102

At -KRNAQLCVLAEDCNQPDYVKLVKALC--ADHSIKLLTVPSAKTLGEWAGLCKID---SE 110

Tb -RRTAHLCVLADDCEDEEYKKLVTALAK--QNNIDLVSMDEREKLAQWAGLTRMA---AD 112

Ec -ADKMSFVMVAENA-EPRISKLVMALAK--KKNIPVISIGSCLELGRIVGVENVS---SS 97

Sv ATSNVKVILLANDCKEAGIKNLVKALAK--QHSIGVCEKFGAAHLGELAHQYVIKGHVTE 91

Hs GKPRKVVGCSCVVVKDYGKESQAKDVIEEYFKCKK--- 132

Sc GNARKVVGASVVVVKNWGAETDELSMIMEHFSQQ---- 143

Dd LAARKVVACSTLVIKTFGKESDDYKFLMEYISKQ---- 136

At GNARKVVGCSCLVIKDFGEETTALNIVKKHLDSN---- 144

Tb GSVRKTLKCSCLAVRDFGERTKALDYLLSQLQ------ 144

Ec GKVR-SKGCCVAGVQDYCEQTSEAGFVQAALLKGISSQ 134

Sv GKIGKVRNASCMAIQNFGTLTAEDQAAFNALLQ----- 124

**(E) Ribosomal protein S24 (RP S24)**

Hs ----MN-DTVTIRTRKFMTNRLLQRKQMVIDVLHPGKA-TVPKTEIREKLAKMYKTTP-D 53

Dm ----MSGTTATIRTRKFMTNRLLARKQMVCDVLHPGLS-SVNKTEIREKLAAMYKVTP-D 54

Sc ----MSD-AVTIRTRKVISNPLLARKQFVVDVLHPNRA-NVSKDELREKLAEVYKAEK-D 53

At ----MAEKAVTIRTRKFMTNRLLSRKQFVIDVLHPGRA-NVSKAELKEKLARMYEVKDPN 55

Pm ------MAEFTVRTRKFITNPLLGRKQFVVDVLHPGVG-SVSKKDLADSLAKMYKVKDAR 53

Tt -------MTIVIRTKKILVNPLLSRRQLSLDVLHPDSP-TASKEKIREELAKQLKVDA-R 51

Tp ----MSDQSVVVKTRKFMKNPLLARRQMIVDIIHPGRA-NVAKSELQEVVGGMHKTDS-K 54

Gl ------MPEITVKVRKVLNNPLLQRQQCVVDVLHPGCT-YESKEAIKAKVAQQLKVADQK 53

Sv ---------MQIKYREIVNNPILDRTQMKLKIVHPGKS-VGTIEALRELVQKDRKIKDIK 50

Sv (with intron)---------MQIKYREIVNNPILDRTQMVSLNLMYNQYQQVRSSRSSTQVSPWVPSRLSA

Hs VIFVFGFRTHFGGGKTTGFGMIYDSLDYAKKNEPKHRLARHGLYEKKKT-SRKQRKERKN 112

Dm VVFAFGFRTNFGGGRSTGFALIYDTLDFAKKFEPKYRLARHGLFEQKKQ-TRKQRKERRN 113

Sc AVSVFGFRTQFGGGKSVGFGLVYNSVAEAKKFEPTYRLVRYGLAEKVEKASRQQRKQKKN 113

At AIFVFKFRTHFGGGKSSGFGLIYDTVESAKKFEPKYRLIRNGLDTKIEK-SRKQIKERKN 114

Pm VISLFGFKTQFGGGRSTGFGLIYDTVEKAQAFEPKHRLRRHGLAP-EFQAKRRSYKELKN 112

Tt NVVVYGFSTQYGGGKSTGFALVYDNQQYLLKYEPNYRLRKVKILGEKPN-TRRSFKELKR 110

Tp LVVLFGFRTKFGGGKSTGFCVIYDNEDALRKFEPKHRLVRLGLEDKKDR-SRKAMKEAKN 113

Gl NIVLYGFKTSFGGGHTVGFCNAYQNMDALMKYEPGFRKIRCGLIEAPKPVSRKQLKNLKN 113

Sv QVVVFDCHTKHGGNLSTASCHIYGNVETLKKVEPKYTIIRLGYIEKPKPVSRKMIKNHKN 110

Sv (with intron)SSSRRIVRSRTSSRLSSLTATPSTVVTSALLLATSTATLRP*

Hs RMKKVRGTAKANVGAGKKPKE------------------ 133

Dm RMKKVRGTAKAKIGTGKK--------------------- 131

Sc RDKKIFGTGKRLAKKVARRNAD----------------- 135

At RAKKIRGVKKTKAGDAKKK-------------------- 133

Pm KCKKVRGTAKSKLR-SK---------------------- 128

Tt KIKRTSGKAITKLLSEKKGDTWASVQSKKSDHLKNFVAK 149

Tp KGKKTRGTGASVAKHKAKRAANSD--------------- 137

Gl RRLKKRGTEKATVTLGAKK-------------------- 132

Sv KLIRKFGTAKSKIVMSGKKN------------------- 130
