# Supplementary material for: Patterns of conservation of spliceosomal intron structures and spliceosome divergence in representatives of the diplomonad and parabasalid lineages
Source: BMC Evol Biol. 2019 Aug 2;19:162. doi: 10.1186/s12862-019-1488-y (PMC6679479; doi:10.1186/s12862-019-1488-y)
Supplement: Supplementary file 4 — ClustalW2 alignment of S. vortens gene alleles containing intron sequences. This file contains nucleotide alignments for all intron-containing RP gene sequences found within S. vortens raw genomic sequence reads and readily identifies several unique RP gene alleles. (DOCX 20 kb) [file 12862_2019_1488_MOESM4_ESM.docx]

**Additional File 4 - ClustalW2 alignment of *S. vortens* gene alleles containing intron sequences.**

Genomic trace database sequences were searched on the NCBI website using blastn and all sequences encoding *S. vortens* gene paralogs containing introns were obtained. Intron sequences and their flanking upstream and downstream exonic sequences were then aligned using ClustalW2. Unique gene paralog sequences are indicated in differential highlighting with intron sequences in red text. Only as single genomic trace for the *Rpl30* gene was identified and thus was excluded from the alignments.

**A) Ribosomal Protein L7a (*Rpl7a*)**

gnl|ti|2141614280_[429_117] AGAAGGAGGCCAAGCTGGTCCTCATCGCCCACGACGTCGACCCAATCGAG 50

gnl|ti|2141629602_[361_673] AGAAGGAGGCCAAGTTGGTCCTCATCGCCCACGACGTCGACCCAATCGAG 50

gnl|ti|2141515448_[326_638] AGAAGGAGGCCAAGCTGGTCCTCATCGCCCACGACGTCGACCCAATCGAG 50

gnl|ti|2141473674_[1022_710] GAAAGGAGGCCAAGCTGTTCTTCATCGCCCACGACGTCGACCCAATCGAG 50

************ ** ** *****************************

gnl|ti|2141614280_[429_117] GTAAGTCTAAACTAACTGTGATCCGCGGATCGCTTGAGTTACGAAACTTT 100

gnl|ti|2141629602_[361_673] GTAAGTCTAAACTAACTGTGATCCGCGGATCGCTTGAGTTACGAAACTTT 100

gnl|ti|2141515448_[326_638] GTAAGTCTAAACTAACTGTGATCCGCGGATCGCTTGAGTTACGAAACTTT 100

gnl|ti|2141473674_[1022_710] GTAAGTCTAAACTAACTGTGATCCGCGGATCGCTGAGTTTACGAAACTTT 100

********************************** ************

gnl|ti|2141614280_[429_117] GCTAACAAACTAGCTCGTCCTGTACCTGCCAACCCTCTGCCACAAGAACA 150

gnl|ti|2141629602_[361_673] GCTAACAAACTAGCTCGTCCTGTACCTGCCAACCCTCTGCCACAAGAACA 150

gnl|ti|2141515448_[326_638] GCTAACAAACTAGCTCGTCCTGTACCTGCCAACCCTCTGCCACAAGAACA 150

gnl|ti|2141473674_[1022_710] GCTAACAAACTAGCTCGTCCTGTACCTGCCAACCCTCTGCCACAAGAACA 150

**************************************************

gnl|ti|2141614280_[429_117] ACATCCCATATGCCATCGTTCGCTCCCGCACCGAGCTCGGCAAGCTGGTT 200

gnl|ti|2141629602_[361_673] ACATCCCTTATGCCATCGTTCGCTCCCGCACCGAGCTCGGCAAGCTGGTT 200

gnl|ti|2141515448_[326_638] ACATCCCATATGCCATCGTTCGCTCCCGCACCGAGCTCGGCAAGCTGGTT 200

gnl|ti|2141473674_[1022_710] ACATCCCATATGCCATCGTTCGCTCCCGCACGAAGCTCGGCAAGCTGGTT 200

******* *********************** *****************

gnl|ti|2141614280_[429_117] CACTGCACCAAGTGCACCTCCATCGCCTTCACCACCATCAAGCCGGAGGA 250

gnl|ti|2141629602_[361_673] CACTGCACCAAGTGCACCTCCATCGCCTTCACCACCATCAAGCCGGAGGA 250

gnl|ti|2141515448_[326_638] CACTGCACCAAGTGCACCTCCATCGCCTTCACCACCATCAAGCCGGAGGA 250

gnl|ti|2141473674_[1022_710] CACTGCACCAAGTGCACCTCCATCGCCTTCACCACCATCAAGCCGGAGGA 250

**************************************************

gnl|ti|2141614280_[429_117] CACCGCCGCCTTCAAGTCCATCCTGGACACCGTCGCCCACGAGGTCGACT 300

gnl|ti|2141629602_[361_673] CACCGCCGCCTTCAAGTCCATCCTGGACACCGTCGCCCACGAGGTCGACT 300

gnl|ti|2141515448_[326_638] CACCGCCGCCTTCAAGTCCATCCTGGACACTGTTGCCCACGAGGTCGACT 300

gnl|ti|2141473674_[1022_710] CACCGCCGCCTTCAAGTCCATCCTGGACACTGTTGCCCACGAGGTCGACT 300

****************************** ** ****************

gnl|ti|2141614280_[429_117] ACGTCCACGCCAT 313

gnl|ti|2141629602_[361_673] ACGTCCACGCCAT 313

gnl|ti|2141515448_[326_638] ACGTCCACGCCAT 313

gnl|ti|2141473674_[1022_710] ACGTCCACGCCAT 313

*************

**B) Ribosomal Protein S4 (*Rps4*)**

gnl|ti|2141479638_[515_772] TTTTTTACATAAAAGTGTGAAAAATATA-C---AGTT------TTGTACA 40

gnl|ti|2141550682_[26_283] -TTTTTACATAAAAGTGTGAAAAATATNAC---AGTT------TTGTACA 40

gnl|ti|2141495195_[88_345] -TTATAATGCAGAAATATGAAAA-TATAACTAGAATCATTATATCATACA 48

gnl|ti|2141536103_[796_539] –TTATAATGCAGAAATATGAAAA-TATAACTAGAATCATTAGATCATACA 48

** * * * ** * ****** *** * * * * ****

gnl|ti|2141479638_[515_772] AATAAAATATTTATATAAATACTATATGTTTCACACCTTTTCTTTTGTTG 90

gnl|ti|2141550682_[26_283] AATAAAATATTTATATAAATACTATATGTTTCACACCTTTTCTTTTGTTG 90

gnl|ti|2141495195_[88_345] TAGAAAAT-TTCATAT---CACGAT---TTCCACACCTTTTCTTTTGATA 91

gnl|ti|2141536103_[796_539] TAGATAAT-TTCACGT---CACGAT---TTCCACACCTTTTCTTTTGATA 91

* * *** ** * * ** ** ** **************** *

gnl|ti|2141479638_[515_772] TGATAAC**ATG**GTAAGTCTAAAATGTGTGCGCACGGCGCATCATCTATTTG 140

gnl|ti|2141550682_[26_283] TGATAAC**ATG**GTAAGTCTAAAATGTGTGCGCACGGCGCATCATCTATTTG 140

gnl|ti|2141495195_[88_345] TC-TATT**ATG**GTAAGTCTAAAATGTGTGCGCACGCCGCATCATATATTTT 140

gnl|ti|2141536103_[796_539] TC-TATT**ATG**GTAAGTCTAAAATGTGTGCGCACGCCGCATCATATATTTT 140

* ** *************************** ******** *****

gnl|ti|2141479638_[515_772] CTTGAACTAACAAACTAGGCTCGTGGTCCAAAACTTCATATGAAACGTCT 190

gnl|ti|2141550682_[26_283] CTTGAACTAACAAACTAGGCTCGTGGTCCAAAACTTCATATGAAACGTCT 190

gnl|ti|2141495195_[88_345] CTTGAACTAACAAGCTAGGCTCGTGGTCCAAAACTTCATATGAAACGCCT 190

gnl|ti|2141536103_[796_539] CTTGAACTAACAAGCTAGGCTCGTGGTCCAAAACTTCATATGAAACGCCT 190

************* ********************************* **

gnl|ti|2141479638_[515_772] TAACGCTCCATCCCACTGGCAGCAGGACAAGCTTGGCGGCATCTACTCCA 240

gnl|ti|2141550682_[26_283] TAACGCTCCATCCCACTGGCAGCAGGACAAGCTTGGCGGCATCTACTCCA 240

gnl|ti|2141495195_[88_345] TAACGCTCCATCCCACTGGTAGCAGGACAAGCTTGGTGGCATTTACTCCA 240

gnl|ti|2141536103_[796_539] TAACGCTCCATCCCACTGGTAGCAGGACAAGCTTGGTGGCATTTACTCCA 240

******************* **************** ***** *******

gnl|ti|2141479638_[515_772] CCAAGTGCAACCTCTCCA 258

gnl|ti|2141550682_[26_283] CCAAGTGCAACCTCTCCA 258

gnl|ti|2141495195_[88_345] CCAAGTGCAACCTCTCCA 258

gnl|ti|2141536103_[796_539] CCAAGTGCAACCTCTCCA 258

******************

**C) Ribosomal Protein S12 (*Rps12*)**

gnl|ti|2141614914_[152_392] TCCACATCCTGAGACAGCATTTATCGAACTATAATTTTGTCTAAATTTAA 50

gnl|ti|2141634934_[500_260] TCCACATCCTGAGACAGCATTTATCGAACTATAATTTTGTCTAAATTTAA 50

gnl|ti|2141503180_[211_451] --CCCATCCTGAGACAGCATTTATCGAACTATAATTTTGTCTAAATTTAA 48

gnl|ti|2141498925_[552_792] -CCACATCCTGAGACAGCATTTATCGAACTATAATTTTGTCTAAATTTAA 49

gnl|ti|2141599697_[595_355] -CCACATCCTGAGACAGCATTTATCGAACTATAATTTTGTCTAAATTTAA 49

* **********************************************

gnl|ti|2141614914_[152_392] –TGAATATCC-AAATAATTTCCATACCTTTTCTTGTGAAGATACATGTCA 98

gnl|ti|2141634934_[500_260] –TGAATATCC-AAATAATTTCCATACCTTTTCTTGTGAAGATACATGTCA 98

gnl|ti|2141503180_[211_451] ATGAATATCCCAAATAATTTCCATACCTTTTCTTGTGAAGATACATGTCA 98

gnl|ti|2141498925_[552_792] ATGAATATCC-AAATAATTTCCATACCTTTTCTTGTGAAGATACATGTCA 98

gnl|ti|2141599697_[595_355] ATGAATATCC-AAATAATTTCCATACCTTTTCTTGTGAAGATACATGTCA 98

********* ***************************************

gnl|ti|2141614914_[152_392] ACGTAAGTCTAGAGCTGAGCAGTCAACTTTACTAACAAAATAGTGACCAA 148

gnl|ti|2141634934_[500_260] ACGTAAGTCTAGAGCTGAGCAGTCAACTTTACTAACAAAATAGTGACCAA 148

gnl|ti|2141503180_[211_451] ACGTAAGTTTAGAGCTGAGCAGTCAACTTTACTAACAAAATAGTGACCAA 148

gnl|ti|2141498925_[552_792] ACGTAAGTCTAGAGCTGAGCAGTCAACTTTACTAACAAAATAGTGACCAA 148

gnl|ti|2141599697_[595_355] ACGTAAGTCTAGAGCTGAGCAGTCAACTTTACTAACAAAATAGTGACCAA 148

******** *****************************************

gnl|ti|2141614914_[152_392] CTGAAGACTTTCTGCAAGAAGATCCGCGTCCACGGCGCGATGGTCTCCGG 198

gnl|ti|2141634934_[500_260] CTGAAGACTTTCTGCAAGAAGATCCGCGTCCACGGCGCGATGGTCTCCGG 198

gnl|ti|2141503180_[211_451] CTGAAGACTTTCTGCAAGAAGATCCGCGTCCACGGCGCGATGGTCTCCGG 198

gnl|ti|2141498925_[552_792] CTGAAGACTTTCTGTAAGAAGATCCGCGTCCACGGTGCTATGGTCTCCGG 198

gnl|ti|2141599697_[595_355] CTGAAGACTTTCTGTAAGAAGATCCGCGTCCACGGTGCTATGGTCTCCGG 198

************** ******************** ** ***********

gnl|ti|2141614914_[152_392] CGTCCGCCAGGTCGTGCGCGCCGTCGAGAACCACGCCACCTCC 241

gnl|ti|2141634934_[500_260] CGTCCGCCAGGTCGTGCGCGCCGTCGAGAACCACGCCACCTCC 241

gnl|ti|2141503180_[211_451] CGTCCGCCAGGTCGTGCGCGCCGTCGAGAACCACGCCACCTCC 241

gnl|ti|2141498925_[552_792] CGTCCGCCAGGTCGTGCGCGCCGTCGAGAACCACGCCACCTCC 241

gnl|ti|2141599697_[595_355] CGTCCGCCAGGTCGTGCGCGCCGTCGAGAACCACGCCACCTCC 241

*******************************************

**D) Ribosomal Protein S24 (*Rps24*)**

gnl|ti|2141541737_[73_313] ATTATTCAAACGAATGTTTTCTTATCTTTTCTTTTGTGGCCTAATGCAGA 50

gnl|ti|2141586865_[578_818] ATTATTCAAACGAATGTTTTCTTATCTTTTCTTTTGTGGCCTAATGCAGA 50

gnl|ti|2141602726_[494_254] ATTATTCAAACGAATGTTTTCTTATCTTTTCTTTTGTGGCCTAATGCAGA 50

gnl|ti|2141597815_[404_164] ATTATTCAAACGAATGTTTTCTTATCTTTTCTTTTGTGGCCTAATGCAGA 50

**************************************************

gnl|ti|2141541737_[73_313] TCAAGTATCGCGAAATTGTCAACAACCCGATCCTCGATCGTACTCAAATG 100

gnl|ti|2141586865_[578_818] TCAAGTATCGCGAAATTGTCAACAACCCGATCCTCGATCGTACTCAAATG 100

gnl|ti|2141602726_[494_254] TCAAGTATCGCGAAATTGTCAACAACCCGATCCTCGATCGTACTCAAATG 100

gnl|ti|2141597815_[404_164] TCAAGTATCGCGAAATTGTCAACAACCCGATCCTCGATCGTACTCAAATG 100

**************************************************

gnl|ti|2141541737_[73_313] GTAAGTCTAAATCTCATGTATAACTAATACTAACAAGTTAGAAGCTCAAG 150

gnl|ti|2141586865_[578_818] GTAAGTCTAAATCTCATGTATAACTAATACTAACAAGTTAGAAGCTCAAG 150

gnl|ti|2141602726_[494_254] GTAAGTCTAAATCTCATGTATAACTAATACTAACAAGTTAGAAGCTCAAG 150

gnl|ti|2141597815_[404_164] GTAAGTCTAAATCTCATGTATAACTAATACTAACAAGTTAGAAGCTCAAG 150

**************************************************

gnl|ti|2141541737_[73_313] ATCGTCCACCCAGGTAAGTCCGTGGGTACCATCGAGGCTCTCCGCGAGCT 200

gnl|ti|2141586865_[578_818] ATCGTCCACCCAGGTAAGTCCGTGGGTACCATCGAGGCTCTCCGCGAGCT 200

gnl|ti|2141602726_[494_254] ATCGTCCACCCAGGTAAGTCCGTGGGTACCATCGAGGCTCTCCGCGAGCT 200

gnl|ti|2141597815_[404_164] ATCGTCCACCCAGGTAAGTCCGTGGGTACCATCGAGGCTCTCCGCGAGCT 200

**************************************************

gnl|ti|2141541737_[73_313] CGTCCAGAAGGATCGTAAGATCAAGGACATCAAGCAGGTTG 241

gnl|ti|2141586865_[578_818] CGTCCAGAAGGATCGTAAGATCAAGGACATCAAGCAGGTTG 241

gnl|ti|2141602726_[494_254] CGTCCAGAAGGATCGTAAGATCAAGGACATCAAGCAGGTTG 241

gnl|ti|2141597815_[404_164] CGTCCAGAAGGATCGTAAGATCAAGGACATCAAGCAGGTTG 241

*****************************************

**E) Bifunctional folylpolyglutamate synthase-like gene (*FolC-like*)**

gnl|ti|2141479887_[559_319] ACTGATAAGAAAAATTAATATGTTACTATTTAACTTTATTACGCATATAA 50

gnl|ti|2141588169_[695_455] ACTGATAAGAAAAATTAATATGTTACTATTTAACTTTATTACGCATATAA 50

gnl|ti|2141538557_[649_889] ACTGATAAGAAAAATTAATATGTTACTATTTAACTTTATTACGCATATAA 50

gnl|ti|2141671053_[509_749] ACTGATAAGAAAAATAAATATGTTGCTATTTAACTTTATTATGGATATAA 50

gnl|ti|2141610787_[847_607] CNTGATAAGAAAAATAAATATGTTACTATTTAACTTTATAATGTATATAA 50

************* ******** ************** * * ******

gnl|ti|2141479887_[559_319] ATTAAGAATTTCACTATTCGACATTAACG**ATG**TAGTACCCTCAAGTGCTT 100

gnl|ti|2141588169_[695_455] ATTAAGAATTTCACTATTCGACATTAACGATGTAGTACCCTCAAGTGCTT 100

gnl|ti|2141538557_[649_889] ATTAAGAATTTCACTATTCGACATTAACGATGTAGTACCCTCAAGTGCTT 100

gnl|ti|2141671053_[509_749] ATTGAACAATTCACTATTCGACATTCACGATGTAGTACCCTCAAGTCCTT 100

gnl|ti|2141610787_[847_607] ATTGAAAAATTCACTATTCGACATTAACGATGTAGTACCCTCAAGTGCTT 100

*** * * **************** ******************** ***

gnl|ti|2141479887_[559_319] GTAAGTCAACTTTTGCCATCAAACTTTTGCTAACAAATTAGGATTCCCTC 150

gnl|ti|2141588169_[695_455] GTAAGTCAACTTTTGCCATCAAACTTTTGCTAACAAATTAGGATTCCCTC 150

gnl|ti|2141538557_[649_889] GTAAGTCAACTTTTGCCATCAAACTTTTGCTAACAAATTAGGATTCCCTC 150

gnl|ti|2141671053_[509_749] GTAAGTCAACTTTTGTCATCAAACTTTTGCTAACAAATTAGGATTCCCTC 150

gnl|ti|2141610787_[847_607] GTAAGTCAACTTTTGTCATCAAACTTTTGCTAACAAATTAGGATTCCATC 150

*************** ******************************* **

gnl|ti|2141479887_[559_319] TCAAAAGTATCATAAACTGTCAAGTCGCATTGGTCATAGCTACCAGATCT 200

gnl|ti|2141588169_[695_455] TCAAAAGTATCATAAACTGTCAAGTCGCATTGGTCATAGCTACCAGATCT 200

gnl|ti|2141538557_[649_889] TCAAAAGTATCATAAACTGTCAAGTCGCATTGNTCATAGCTACCAGATCT 200

gnl|ti|2141671053_[509_749] TCANAAGTATCATAAACTGTTAAGTCGCATTGGTCATAGCTACCAGATCT 200

gnl|ti|2141610787_[847_607] TCAAAAGTATCATAAACTGTCAAGTCGCATTGGTCATAGCTACCAGATTT 200

*** **************** *********** *************** *

gnl|ti|2141479887_[559_319] ACTAAATAAGTAATTTCGACCGGAATTGCTGTTTCACGTCA 241

gnl|ti|2141588169_[695_455] ACTAAATAAGTAATTTCGACCGGAATTGCTGTTTCACGTCA 241

gnl|ti|2141538557_[649_889] ACTANATAAGTAATTTCGACCGGGATTGCTGTTTCACGTCA 241

gnl|ti|2141671053_[509_749] ACTAAATAAGTAATTTCGACCGGAATTGCTGTTTCACGTCA 241

gnl|ti|2141610787_[847_607] ACTAAATAAGTAATTTCGACCGGAATTGCTGTTTCACGTCA 241

**** ****************** *****************

**F) Hypothetical ORF #1**

gnl|ti|2141628303_[520_739] -TTGTATTGGTAATGATACAATATATTGAAATATTTTCGTTTCCTCCGAA 49

gnl|ti|2141664662_[809_509] -TTGTATTGGTAATGATACAATATATTGAAATATNTTCGTTTCCTCCGAA 49

gnl|ti|2141612529_[78_297] -TTGTATTGGTAATGATACAATATATTGAAATATTTTCGTTTCCTCCGAA 49

gnl|ti|2141491491_[416_197] -TTGTATTGGTAATGATACAATATATTGAAATATTTTCGTTTCCTCCGAA 49

gnl|ti|2141541942_[483_702] -TTGCATTGGTAATGATACAATATATTGTAATATTTTCGTTTCCTCCGAA 49

gnl|ti|2141510109_[430_211] -TTGCATTGGTAATGATACAATATATTGTAATATTTTCGTTTCCTCCGAA 49

gnl|ti|2141636533_[463_682] -TTGCATTGGTAATGATACAATATATTGTAATATTTTCGTTTCCTCCGAA 49

gnl|ti|2141657616_[782_945] ATTGCATTGGTAATGATACCATATATTGTAATATTTTCGTTTCCTC-GAA 49

gnl|ti|2141510109_[228_9] -TTGCATTGGTAATGATACAATATATTGTAATATTTTCGTTTCCTCCGAA 49

*** ************** ******** ***** *********** ***

gnl|ti|2141628303_[520_739] TGTCCGAAACCTCGTCCTCCAGCGACGCTGGAGACGCTTTCGAGTAATAT 99

gnl|ti|2141664662_[809_509] TGTCCGAAACCTCGTCCTCCAGCGACGCTGGAGACGCTTTCGAGTAATAT 99

gnl|ti|2141612529_[78_297] TGTCCGAAACCTCGTCCTCCAGCGACGCTGGAGACGCTTTCGAGTAATAT 99

gnl|ti|2141491491_[416_197] TGTCCGAAACCTCGTCCTCCAGCGACGCTGGAGACGCTTTCGAGTAATAT 99

gnl|ti|2141541942_[483_702] TGTCCGAAACCTCGTCCTCCAGCGACGCTGGAGACGCTTTCGAGTAATAT 99

gnl|ti|2141510109_[430_211] TGTCCGAAACCTCGTCCTCCAGCGACGCTGGAGACGCTTTCGAGTAATAT 99

gnl|ti|2141636533_[463_682] TGTCCGAAACCTCGTCCTCCAGCGACGCTGGAGACGCTTTCGAGTAATAT 99

gnl|ti|2141657616_[782_945] TGTCCGAAACCTCGTCCTCCAGCGACGCTGGAGACGCTTTCGAGTAATAT 99

gnl|ti|2141510109_[228_9] TGTCCGAAACCTCGTCCTCCAGCGACGCTGGAGACGCTTTCGAGTAATAT 99

**************************************************

gnl|ti|2141628303_[520_739] CGTAGGTCTAATTGATATTGATAACTTTACTAACAAACTAGTGCAATAGC 149

gnl|ti|2141664662_[809_509] CGTAGGTCTAATTGATATTGATAACTTTACTAACAAACTAGTGCAATAGC 149

gnl|ti|2141612529_[78_297] CGTAGGTCTAATTGATATTGATAACTTTACTAACAAACTAGTGCAATAGC 149

gnl|ti|2141491491_[416_197] CGTAGGTCTAATTGATATTGATAACTTTACTAACAAACTAGTGCAATAGC 149

gnl|ti|2141541942_[483_702] CGTAGGTCTAATTGATATGGATAACTTTACTAACAAACTAGTGCAATAGC 149

gnl|ti|2141510109_[430_211] CGTAGGTCTAATTGATATGGATAACTTTACTAACAAACTAGTGCAATAGC 149

gnl|ti|2141636533_[463_682] CGTAGGTCTAATTGATATGGATAACTTTACTAACAAACTAGTGCAATAGC 149

gnl|ti|2141657616_[782_945] CGTAGGTCTAAATGATATGGATAACTTTACTAACAAACTAGTGCAATAGC 149

gnl|ti|2141510109_[228_9] CGTAGGTCTAATTGATATGGATAACTTTACTAACAAACTAGTGCAATAGC 149

*********** ****** *******************************

gnl|ti|2141628303_[520_739] GCCAGAAAAAGAACCAAGAAATCGAAGAATTAAAGAATCAAACGCAGTAA 199

gnl|ti|2141664662_[809_509] GCCAGAAAAAGAACCAAGAAATCGAAGAATTAAAGAATCAAACGCAGTAA 199

gnl|ti|2141612529_[78_297] GCCAGAAAAAGAACCAAGAAATCGAAGAATTAAAGAATCAAACGCAGTAA 199

gnl|ti|2141491491_[416_197] GCCAGAAAAAGAACCAAGAAATCGAAGAATTAAAGAATCAAACGCAGTAA 199

gnl|ti|2141541942_[483_702] GCCAGAAAAAGAACCAAGAAATCGAAGAATTAAAGAATCAAACGCAGTAA 199

gnl|ti|2141510109_[430_211] GCCAGAAAAAGAACCAAGAAATCGAAGAATTAAAGAATCAAACGCAGTAA 199

gnl|ti|2141636533_[463_682] GCCAGAAAAAGAACCAAGAAATCGAAGAATTAAAGAATCAAACGCAGTAA 199

gnl|ti|2141657616_[782_945] GCCCAGAAAAGAACC----------------------------------- 164

gnl|ti|2141510109_[228_9] GCCAGAAAAAGAACCAAGAAATCGAAGAATTAAAGAATCAAACGCAG-AA 198

*** *********
