# Supplementary material for: Patterns of conservation of spliceosomal intron structures and spliceosome divergence in representatives of the diplomonad and parabasalid lineages
Source: BMC Evol Biol. 2019 Aug 2;19:162. doi: 10.1186/s12862-019-1488-y (PMC6679479; doi:10.1186/s12862-019-1488-y)
Supplement: Supplementary file 5 — Base pairing potential in S. salmonicida introns. This file confirms secondary structural potential for the S. salmonicida Rpl30 intron and an alignment showing the length distributions for the four known S. salmonicida introns (Xu et al. 2014). (DOCX 74 kb) [file 12862_2019_1488_MOESM5_ESM.docx]

**Additional File 5 - Base pairing potential in the *S. salmonicida Rpl30* intron.**

**(A)** MFOLD secondary structural prediction for the *Rpl30* intron from *S. salmonicida* (Xu *et al.* 2014) is shown as described in Figure 2, with the predicted single stranded length indicated. **(B)** ClustalW2 alignment of *S. salmonicida* introns (modified from Xu *et al.* 2014), with stem loop forming nucleotides from the *Rpl30* intron in red and total intron lengths indicated in nucleotides (nt).

**(A)**


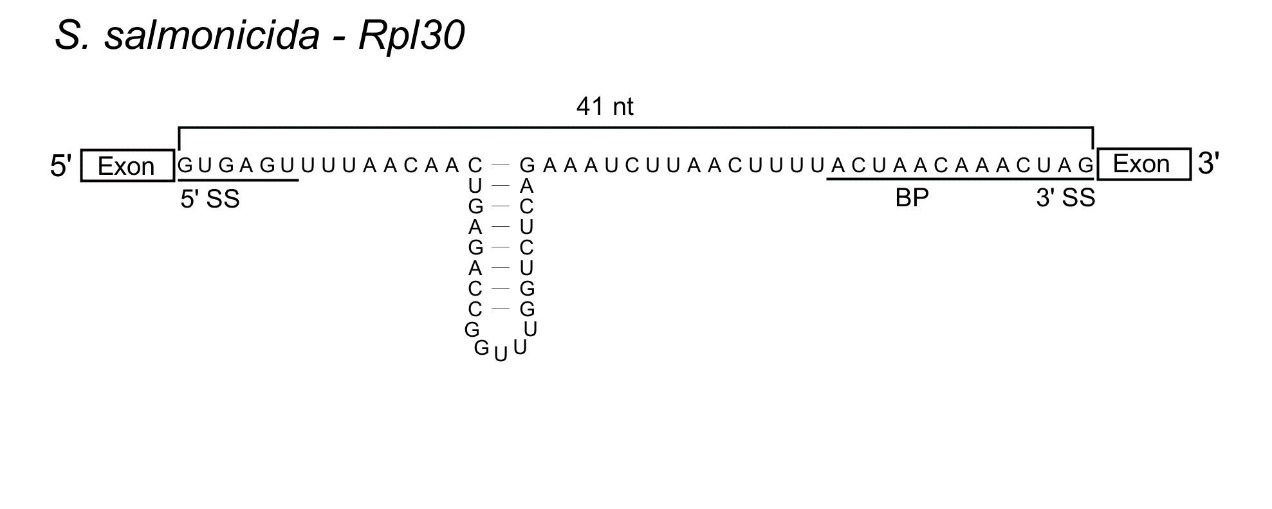


**(B)**

SS5-377_16979(*Rpl30*) GUGAGUUUUAACAACUGAGACCGGUUUGGUCUCAGAAAUCUUAACUUUUACUAACAAACUAG 67 nt

SS5-377_16134 GUAUGUUUUAA-------------------CAAUUAAAAAAUAACUUAUACUAACAAACUAG 43 nt

SS5-377_18398 GUAUGUUUUAA-------------------CUCAAUAAAUACAACUUUUACUAACAAACUAG 43 nt

SS5-377_17358 GUAUGUCUAAA-------------------CUUUUUUAAUGUAACUUAUACUAACAAACUAG 43 nt

** ** * ** * * ***** **************
