# Supplementary material for: Patterns of conservation of spliceosomal intron structures and spliceosome divergence in representatives of the diplomonad and parabasalid lineages
Source: BMC Evol Biol. 2019 Aug 2;19:162. doi: 10.1186/s12862-019-1488-y (PMC6679479; doi:10.1186/s12862-019-1488-y)
Supplement: Supplementary file 6 — Structural potential of cis-spliceosomal introns in Trichomonas vaginalis. This file contains a table showing the collection of known T. vaginalis spliceosomal introns and regions of introns predicted to form stem-loops by MFOLD software. Predicted single stranded distances between splice sites are also shown. (DOCX 18 kb) [file 12862_2019_1488_MOESM6_ESM.docx]

**Additional File 6 - Structural potential of *cis*-spliceosomal introns in *Trichomonas vaginalis*.**

Spliceosomal introns sequences from *T. vaginalis* genes are shown with red text indicating intron regions predicted to form secondary structure by MFOLD software (Zuker 2003). Total intron lengths and the predicted single stranded (SS) length of folded pre-mRNA introns are indicated.

| **Gene ID** | **Genomic Location** | **Gene Description** | **Intron Sequence** | **Intron Length** | **Intron SS Length** |
| --- | --- | --- | --- | --- | --- |
| TVAG_014960 | DS113774: 39,976 - 41,345 (+) | TATA binding protein associated factor, putative | gtacgtatttctttggtttctggcttgttttattttaaaataaaccaggaaccaaattttttttcagaaactaacacacag | 81 | 36 |
| TVAG_020880 | DS113200: 11,125 - 13,510 (-) | AGC family protein kinase | gtatgtattttttattttttcatcgattacaaaattttttctgaaaatataagatttgagatatatatttcgaataccgaaaattttgatgatttttttatgaaattttttggtattttttttatatctcaaaaattatttcagaaaaaaagaaagtggttgatgaaataatttttagatcattactaacacacag | 196 | 37 |
| TVAG_043580 | DS113505: 33,566 - 34,321 (-) | maintenance of ploidy protein mob2, putative | gtacagttttaattctaacaaacag | 25 | 25 |
| TVAG_053820 | DS113785: 33,670 - 35,267 (+) | CAMK family protein kinase | gtatgttttttaatgaaattttaataacaaaaaaaaattttcaaaaattccaaattttttttgttattaaaatttcatattttttttattttaaaaatactaacacacag | 110 | 24 |
| TVAG_056030 | DS113419: 42,709 - 43,113 (+) | conserved hypothetical protein | gttctatttaatttctaacaaacag | 25 | 25 |
| TVAG_065500 | DS115094: 3,745 - 5,333 (-) | CAMK family protein kinase | gtatgtatttttttggtaaacctcaattttttcaaatgactttcttaacctttcgaaatatactttcaaaagtgttagaaatgtcatattgtaaataagaggtgccatttttgttaacatttactaacacacag | 134 | 39 |
| TVAG_085780 | DS114439: 10,258 - 11,686 (+) | conserved hypothetical protein | gtatgtactttttgagctggtaacattatttaccagctccttttacttagaaaatactaacacacag | 67 | 41 |
| TVAG_087980 | DS113624: 20,938 - 22,047 (-) | STE family protein kinase | gtatgtatacttttgatgtcaattttatttttttaattgacatcattttcttttttatttttagatactaacacacag | 78 | 47 |
| TVAG_089630 | DS114221: 15,715 - 17,109 (-) | AGC family protein kinase | gttcttttttatttctaacaaacag | 25 | 25 |
| TVAG_110020 | DS113198: 45,552 - 46,947 (+) | TATA binding protein associated factor, putative | gtacgtatttttaggcaaagtatttctttttttaatttttaaaaaattgaaatattttgccaaaatttaaacaaaatatactaacacacag | 91 | 43 |
| TVAG_110580 | DS113198: 188,777 - 191,296 (+) | centaurin gamma, putative | gtatgtatattttctggcgtaaaaagaaagatataaattaacttttacccctcatttgcttttaaatgaaagggaatcgtattttatgctttcttttgcgctattttttacatttactaacacacag | 127 | 38 |
| TVAG_125100 | DS113398: 99,086 - 100,336 (+) | CMGC family protein kinase | gtatgttttcgagttcttcgtacataaagaaacagacattttagtctgttttctttttggaaaaaaaaacgaagaattttttaaaaaaaatttactaacacacag | 105 | 36 |
| TVAG_126240 | DS113357: 56,024 - 57,470 (-) | CAMK family protein kinase | gtatgtttcttttattggttatcactattaatagaaaatctcaaaatttttctattaatgtggaaccaaaaacaatttttataagttactaacacacag | 99 | 44 |
| TVAG_130170 | DS113203: 191,775 - 193,935 (+) | conserved hypothetical protein | gtatgtatttatttctaaccaacag | 25 | 25 |
| TVAG_134480 | DS114086: 5,751 - 6,702 (+) | ribosomal protein S6 kinase, 90kD, polypeptide, putative | gttcttttttatttctaacaaacag | 25 | 25 |
| TVAG_147850 | DS114056: 23,260 - 24,449 (-) | CAMK family protein kinase | gtatgtatttttaatttttggagtgttatattgatcattccaatgtttatcatataactaacacacag | 68 | 42 |
| TVAG_148640 | DS113755: 29,291 - 30,330 (+) | CAMK family protein kinase | gtatgtactattttatttttgcctaatttacaaatatttttatttgataaatttcgcaaaaatttttatttttttcaaaatactaacacacag | 93 | 44 |
| TVAG_176980 | DS113680: 30,321 - 31,501 (+) | CMGC family protein kinase | gtacgtatttcatctatgcataatttatgcatagatattttttcaaaactaacacacag | 59 | 34 |
| TVAG_198230 | DS113190: 59,033 - 59,932 (+) | conserved hypothetical protein | gtatgtattttatttcataagttgccaatttttcttttagtataaccggcaacttatgagtttttatacattactaacacacag | 84 | 37 |
| TVAG_217460 | DS113550: 71,345 - 72,565 (-) | ankyrin repeat domain protein, putative | gtatgtacctatttataagcaaattggcgtaacaatacgctatttgcattatttttcaatgctaacacacag | 72 | 34 |
| TVAG_225200 | DS113224: 7,991 - 9,110 (-) | nuclear lim interactor-interacting factor, putative | gtatgtatatttttgtttcatatttcctatttggaaatttgaaaacattttttggaaaaaatttactaacacacag | 76 | 42 |
| TVAG_242770 | DS113657: 12,422 - 13,817 (+) | conserved hypothetical protein | gtactgctttatttctaaccaacag | 25 | 25 |
| TVAG_306990 | DS114021: 8,464 - 9,903 (-) | CMGC family protein kinase | gtatgtttcttacaagcatgtgttcttcgcaatatcgaaattttgcgaagaaaacgtgtttgaaaaaaaaaattaaattttactaacacacag | 93 | 43 |
| TVAG_324910 | DS113569: 18,626 - 19,252 (-) | RNA recognition motif (rrm) domain containing protein, putative | gtacatttttatttctaacaaacag | 25 | 25 |
| TVAG_350500 | DS113477: 60,512 - 62,110 (-) | CAMK family protein kinase | gtatgtacctatttaccttcgataatttcatttaattacgggctaatttagctttttaccgtaattttatgaaattttatcgaaatttttgaaaataattttactaacacacag | 114 | 33 |
| TVAG_360840 | DS113782: 26,243 - 27,435 (-) | ankyrin repeat-cotaining protein, putative | gtatgcattcgaaattcgacttttcagtcgaatgttttttgaatactaacacacag | 56 | 25 |
| TVAG_383350 | DS113985: 30,460 - 31,220 (+) | RAB-2,4,14, putative | gtataatttaatttctaacaaacag | 25 | 25 |
| TVAG_388620 | DS113269: 100,189 - 101,767 (+) | poly(A) polymerase gamma, putative | gtatgtacaatttttttgattaatattattgtttcttgcattttcatgcttgaaacaattatattaattgttttattcattcactaacacacag | 94 | 42 |
| TVAG_390460 | DS113480: 48,186 - 49,227 (-) | nuclear lim interactor-interacting factor, putative | gtatgtataatattacaaatatcttcaatatattttgaagatatttaaattttcaaatactaacacacag | 70 | 40 |
| TVAG_413420 | DS113675: 23,577 - 24,757 (-) | CMGC family protein kinase | gtatgtttcattacacgtctagatttttacgtctagactgtaaatttttttgaattactaacacacag | 68 | 37 |
