# Supplementary material for: Patterns of conservation of spliceosomal intron structures and spliceosome divergence in representatives of the diplomonad and parabasalid lineages
Source: BMC Evol Biol. 2019 Aug 2;19:162. doi: 10.1186/s12862-019-1488-y (PMC6679479; doi:10.1186/s12862-019-1488-y)
Supplement: Supplementary file 7 — Clustal Omega alignments of 5′ RACE and RT-PCR sequencing products with predicted mRNA sequences lacking introns and U2 snRNA. This file contains nucleotide alignments of our 5′ RACE products and U2 snRNA RT-PCR sequencing results with the predicted sequence of RP mRNAs following removal of the proposed intron, and the proposed U2 candidate. (DOCX 17 kb) [file 12862_2019_1488_MOESM7_ESM.docx]

**Additional File 7 – Clustal Omega alignments of 5′ RACE and RT-PCR sequencing products with predicted mRNA sequences lacking introns and U2 snRNA.**

**(A-E)** Predicted introns were removed from genomic trace sequences of RP genes then aligned with sequenced 5′ RACE products using the Clustal Omega web tool. The nucleotides of exons flanking the intron are highlighted, the last 3 nucleotides of the upstream exon are in light blue and the first 3 nucleotides of the downstream exon are in green. ATG start codons are indicated in bold and underlined. **(F)** Sequenced RT-PCR products were aligned with the targeted sequence of the predicted U2 snRNA candidate. For all alignments asterisks indicate identical nucleotides at that position.

**(A)**

Rpl7a AC**ATG**TCTACCGCTGCCAAGCCAGAACACGTCAACAACCTGACCCGCATGGTCAAGTGGC 60

Rpl7a_5′RACE AC**ATG**TCTACCGCTGCCAAGCCAGAACACGTCAACAACCTGACCCGCATGGTCAAGTAGC 60

********************************************************* **

Rpl7a CCGCCTACATCCGCATCCAGCGCCAGAAGGCCCTCCTCCAGCACCGCCTGAAGGTCCCCG 120

Rpl7a_5′RACE CCGCCTACATCCGCATCCAGCGCCAGAAGGCCCTCCTCCAGCACCGCCTGAAGGTCCCCG 120

************************************************************

Rpl7a GCGTCGTCAACATGTTCCGCAACCCGCTGAACGCCAACGCCACCAAGGAGATCCTGAAGT 180

Rpl7a_5′RACE GCGTCGTCAACATGTTCCGCAACCCGCTGAACGCCAACGCCACCAAGGAGATCCTGAAGT 180

************************************************************

Rpl7a TCGCCGCCAAGTACCAGCCGGAGACCAAGGAGGCCAGACAGCAGCGCCTTGTCCAGGCTG 240

Rpl7a_5′RACE TCGCCGCCAAGTACCAGCCGGAGACCAAGGAGGCCAGACAGCAGCGCCTTGTCCAGGCTG 240

************************************************************

Rpl7a CCGACAAGAAGACCACCATCAACGCCCCAGTGTCCTTCAACTACAACATCCACAAGGTTG 300

Rpl7a_5′RACE CCGACAAGAAGACCACCATCAACGCCCCAGTGTCCTTCAACTACAACATCCACAAGGTTG 300

************************************************************

Rpl7a TTGAGGCCGTCGAGAAGAAGGAGGCCAAGTTGGTCCTCATCGCCCACGACGTCGACCCAA 360

Rpl7a_5′RACE TTGAGGCCGTCGAGAAGAAGGAGGCCAAGCTGGTCCTCATCGCCCACGACGTCGACCCAA 360

***************************** ******************************

Rpl7a TCGAGCTCGTCCTGTACCTGCCAACCCTCTGCCACAAGAACAACATCCCTTATGCCATCG 420

Rpl7a_5′RACE TCGAGCTCGTCCTGTACCTGCCAACCCTCTGCCACAAGAACAACATCCCATATGCCATCG 420

************************************************* **********

Rpl7a TTCGCTCCCGCACCGAGCTCGGCAAGCTGGTTCACTGCACCAAGTGCACCTCCATCGCCT 480

Rpl7a_5′RACE TTCGCTCCCGCACCGAGCTCGGCAAGCTGGTTCACTGCACCAAGTGCACCTCCATCGCCT 480

************************************************************

Rpl7a TCACCACCATCAAGCCGGAGGACACCGCCGCCTTCAAGTCCATCCTGGACACCGTCGCCC 540

Rpl7a_5′RACE TCACCACCATCAAGCCGGAGGACACCGCCGCCTTCAAGTCCATCCTGGACACCGTCGCCC 540

************************************************************

**(B)**

Rps15 ACACCTTTTCTTTTGGGTAACTAATTA**ATG**GGTCGTACTAATGTTCTCAATGACGTTCTC 60

Rps15_5′RACE --------------TGGTAACTAATTA**ATG**GGTCGTACTAATGTTCTCAATGACGTTCTC 46

*********************************************

Rps15 AAGCAGATCACCAACGCTCAGCGCCTTGGCAGACGCCAGTGCATCCTGCACC 112

Rps15_5′RACE AAGCAGATCACCAACGCTCAGCGCCTTGGCAGACGCCAGTGCATCCTGCACC 98

****************************************************

**(C)**

Rps24 TGGCCTA**ATG**CAGATCAAGTATCGCGAAATTGTCAACAACCCGATCCTCGATCGTACTCA 60

Rps24_5′RACE -TGCCTA**ATG**CAGATCAAGTATCGCGAAATTGTCAACAACCCGATCCTCGATCGTACTCA 59

**********************************************************

Rps24 AATGAAGCTCAAGATCGTCCACCCAGGTAAGTCCGTGGGTACCATCGAGGCTCTCCGCGA 120

Rps24_5′RACE AATGAAGCTCAAGATCGTCCACCCAGGTAAGTCCGTGGGTACCATCGAGGCTCTCCGCGA 119

************************************************************

Rps24 G 121

Rps24_5′RACE G 120

*

**(D)**

Rpl30 ---**ATG**GATCGCGTATCTAAGAAGTCTTCTGAATCGGCCGCCTTGCAGCTTGCTCTTGTC 57

Rpl30_5′RACE GCA**ATG**GATCGCGTATCTAAGAAGTCTTCTGAATCGGCCGCCTTGCAGCTTGCTCTTGTC 60

*********************************************************

Rpl30 GTCAAGTCCGGCAAGTACACCCTTGGTGTCAACCAGGCTCTTAAGTCCATCCGCAACCTG 117

Rpl30_5′RACE GTCAAGTCCGGCAAGTACACCCTTGGTGTCAACCAGGCTCTTAAGTCCATCCGCAACCTG 120

************************************************************

Rpl30 AAGGCCAAGCTCGTCATCAT 137

Rpl30_5′RACE AAGGCCAAGCTCGTCATCAT 140

********************

**(E)**

Rps4 GTTGTGATAAC**ATG**GCTCGTGGTCCAAAACTTCATATGAAACGTCTTAACGCTCCATCCC 60

Rps4_5′RACE TTTGTGATAAC**ATG**GCTCGTGGTCCAAAACTTCATATGAAACGTCTTAACGCTCCATCCC 60

***********************************************************

Rps4 ACTGGCAGCAGGACAAGCTTGGCGGCATCTACTCCACCAAGTGCAACCTCTCCACCCACA 120

Rps4_5′RACE ACTGGCAGCAGGACAAGCTTGGCGGCATCTACTCCACCAAGTGCAACCTCTCCACCCACA 120

************************************************************

Rps4 GGATCAATGAGTGCGTCCCAATGTCCCTCGTTCTCCGCAACCGCCTGAACCTCGCCAAGA 180

Rps4_5′RACE GGATCAATGAGTGCGTCCCAATGTCCCTCGTTCTCCGCAACCGCCTGAACCTCGCCAAGA 180

************************************************************

**(F)**

U2_RTproduct CAAGTTTCGGCCCTGGTAAAGCAGGGCCTTCCGGTACGCCGGAGCTTCCACTTTTATCAT 60

Predicted_U2_region CAAGTTTCGGCCCTGGTAAAGCAGGGCCTTCCGGTACGCCGGAGCTTCCACTTTTATCAT 60

************************************************************

U2_RTproduct CCGGTCTGGGCCACTCCCTCG 81

Predicted_U2_region CCGGTCTGGGCCACTCCCTCG 81

*********************
