# Supplementary material for: Patterns of conservation of spliceosomal intron structures and spliceosome divergence in representatives of the diplomonad and parabasalid lineages
Source: BMC Evol Biol. 2019 Aug 2;19:162. doi: 10.1186/s12862-019-1488-y (PMC6679479; doi:10.1186/s12862-019-1488-y)
Supplement: Supplementary file 8 — Evolutionary conservation of Rps4 and Rps24 gene introns in eukaryotes. This file contains a table of all organisms examined for rpS4 and rpS24 gene introns. When possible the length and sequences for each identified intron are provided. (DOCX 31 kb) [file 12862_2019_1488_MOESM8_ESM.docx]

**Additional File 8 - Evolutionary conservation of *Rps4* and *Rps24* gene introns in eukaryotes.**

**(A) *Rps4* intron**

|  | **Intron Present?** | **Intron Length (nt)** |
| --- | --- | --- |
| **Archaeaplastida** |  |  |
| **Green Plants (green algae, prasinophytes and land plants)** |  |  |
| **Land plants** |  |  |
| **Eudicots** |  |  |
| Arabidopsis thaliana | 1 | 307 |
| Medicago truncatula | 1 | 1398 |
| Ricinus communis | 1 | 682 |
| Fragaria vesca | 1 | 77 |
| Vitis vinifera | 1 | 937 |
| **Monocots** |  |  |
| Setaria italica | 1 | 845 |
| Brachypodium distachyon | 1 | 740 |
| Oryza sativa | 1 | 670 |
| **Club moss** |  |  |
| Selaginella moellendorffii | 1 | 49 |
| **Moss** |  |  |
| Physcomitrella patens | 1 | 220 |
| **Green algae** |  |  |
| Ostreococcus lucimarinus | 0 |  |
| Chlamydomonas reinhardtii | 0 |  |
| Volvox carteri f. Nagariensis | 0 |  |
| Micromonas pusilla | 0 |  |
| **Rhodophyta (red algae)** |  |  |
| Porphyridium purpureum | 0 |  |
| **Glaucophytes (Cyanophora)** |  |  |
| Cyanophora paradoxa | 0 |  |
| **Total** | 10 |  |
|  |  |  |
|  | **Intron Present?** | **Intron Length (nt)** |
| **Unikonts** |  |  |
| **Opisthokonts** |  |  |
| **Animals (Metazoa)** |  |  |
| **Chordates** |  |  |
| Homo sapiens | 1 | 958 |
| Gallus gallus | 0 |  |
| Oreochromis niloticus | 1 | 563 |
| Xenopus (Silurana) tropicalis | 1 | 675 |
| Ciona intestinalis | 1 | 223 |
| **Arthropods** |  |  |
| Culex quinquefasciatus | 0 |  |
| Drosophila melanogaster | 1 | 95 |
| Bombus impatiens | 1 | 455 |
| Tribolium castaneum | 1 | 338 |
| Bombyx mori | 1 | 316 |
| **Nematodes** |  |  |
| Brugia malayi | 0 |  |
| Trichinella spiralis | 0 |  |
| Loa loa | 0 |  |
| Caenorhabditis elegans | 0 |  |
| **Cnidarians** |  |  |
| Hydra magnipapillata | 1 | 133 |
| **Sponge** |  |  |
| Amphimedon queenslandica | 1 | 80 |
| **Mollusc** |  |  |
| Aplysia californica | 1 | 3183 |
| **Choanoflagellates** |  |  |
| Salpingoeca sp. ATCC 50818 | 0 |  |
| Monosiga brevicollis | 0 |  |
| **Filastera** |  |  |
| Capsaspora owczarzaki ATCC 30864 | 1 | 430 |
| **Ichthyosporea** |  |  |
|  |  |  |
| **Fungi** |  |  |
| **Ascomycetes** |  |  |
| Candida albicans | 0 |  |
| Ashbya gossypii | 0 |  |
| Aspergillus fumigatus | 0 |  |
| Coccidioides posadasii | 0 |  |
| Neurospora crassa | 0 |  |
| Schizosaccharomyces pombe | 0 |  |
| Pyrenophora tritici-repentis | 0 |  |
| Botryotinia fuckeliana | 0 |  |
| Nectria haematococca | 0 |  |
| Magnaporthe oryzae | 0 |  |
| Verticillium albo-atrum VaMs.102 | 0 |  |
| Zymoseptoria tritici | 0 |  |
| **Basidiomycetes** |  |  |
| Coprinopsis cinerea okayama7#130 | 1 | 162 |
| Schizophyllum commune H4-8 | 1 | 143 |
| Cryptococcus neoformans var. neoformans B-3501A | 1 | 151 |
| Postia placenta Mad-698-R | 1 | 210 |
| Puccinia graminis f. sp. tritici CRL 75-36-700-3 | 1 | 147 |
| **Microsporidians** |  |  |
| Encephalitozoon hellem | 0 |  |
| Encephalitozoon intestinalis | 0 |  |
|  |  |  |
| **Amoebozoa** |  |  |
| Physarum polycephalum | 1 | 74 |
| Entamoeba histolytica | 0 |  |
| Entamoeba dispar | 0 |  |
| Entamoeba invadens | 0 |  |
| Acanthamoeba castellanii | 0 |  |
| Dictyostelium discoideum | 1 | 430 |
| Dictyostelium fasciculatum | 0 |  |
| **Total** | 19 |  |
|  |  |  |
|  |  |  |
|  | **Intron Present?** | **Intron Length (nt)** |
| **Excavates** |  |  |
| **Malawimonads** |  |  |
|  |  |  |
| **Euglenozoa** |  |  |
| **Kinetoplastids** |  |  |
| Trypanosoma brucei | 0 |  |
| Trypanosoma cruzi | 0 |  |
| Leishmania braziliensis | 0 |  |
| Leishmania major | 0 |  |
| Leishmania infantum | 0 |  |
| **Heterolobosea** |  |  |
| Naegleria gruberi | 1 | 144 |
| **Jakobida** |  |  |
|  |  |  |
| **Parabasalids** |  |  |
| Trichomonas vaginalis | 0 |  |
| **Fornicata** |  |  |
| Giardia lamblia | 0 |  |
| **Preaxostyla** |  |  |
| **Total** | 1 |  |
|  |  |  |
|  | **Intron Present?** | **Intron Length (nt)** |
| **Chromalveolates** |  |  |
| Cryptomonas paramecium | 0 |  |
| Guillardia theta | 0 |  |
| Hemiselmis andersenii | 0 |  |
| **Rhizaria** |  |  |
| **Cercozoa** |  |  |
| Bigelowiella natans | 0 |  |
| **Foraminifera** |  |  |
|  |  |  |
| **Radiolaria** |  |  |
|  |  |  |
| **Alveolates** |  |  |
| Perkinsus marinus | 0 |  |
| **Apicomplexans** |  |  |
| Theileria parva strain Muguga | 1 | 103 |
| Cryptosporidium muris | 1 | 62 |
| Plasmodium knowlesi | 1 | 675 |
| Babesia equi | 1 | 104 |
| Plasmodium falciparum | 1 | 715 |
| **Ciliates** |  |  |
| Ichthyophthirius multifiliis | 1 | 61 |
|  |  |  |
| **Stramenopiles** |  |  |
| **Diatoms** |  |  |
| Thalassiosira pseudonana | 0 |  |
| Phaeodactylum tricornutum | 0 |  |
| **Oomycetes** |  |  |
| Phytophthora infestans | 0 |  |
| **Total** | 6 |  |
|  |  |  |
| **(B) *Rps24* intron** | | |
|  | **Intron Present?** | **Intron Length (nt)** |
| **Archaeaplastida** |  |  |
| **Green Plants (green algae, prasinophytes and land plants)** |  |  |
| **Land Plants** |  |  |
| **Eudicot** |  |  |
| Glycine max | 1 | 432 |
| Arabidopsis thaliana | 1 | 299 |
| Ricinus communis | 1 | 713 |
| Solanum lycopersicum | 1 | 117 |
| Cucumis sativus | 1 | 321 |
| Fragaria vesca | 1 | 112 |
| Vitis vinifera | 1 | 667 |
| **Monocot** |  |  |
| Sorghum bicolor | 0 |  |
| Setaria italica | 1 | 92 |
| **Club-mosses** |  |  |
| Selaginella moellendorffii | 1 | 73 |
| **Mosses** |  |  |
| Physcomitrella patens | 1 | 137 |
|  |  |  |
| **Green Algae** |  |  |
| Chlamydomonas reinhardtii | 1 | 79 |
| Ostreococcus tauri | 0 |  |
| Ostreococcus lucimarinus | 1 | 168 |
| Volvox carteri | 1 | 71 |
| Micromonas pusilla | 1 | 168 |
|  |  | 0 |
| **Rhodophyta (red algae)** |  |  |
| Porphyridium purpureum | 0 |  |
| **Glaucophytes (Cyanophora)** |  |  |
| Cyanophora paradoxa | 1 | 79 |
| **Total** | 15 |  |
|  |  |  |
|  |  |  |
|  | **Intron Present?** | **Intron Length (nt)** |
| **Unikonts** |  |  |
| **Opisthokonts** |  |  |
| **Animals (Metazoa)** |  |  |
| **Chordates** |  |  |
| Homo sapiens | 1 | 93 |
| Danio rerio | 1 | 106 |
| Gallus gallus | 1 | 327 |
| Anolis carolinensis | 1 | 951 |
| **Arthropods** |  |  |
| Drosophila melanogaster | 0 |  |
| Aedes aegypti | 0 |  |
| Culex quinquefasciatus | 0 |  |
| Apis mellifera | 1 | 167 |
| Nasonia vitripennis | 1 | 75 |
| Pediculus humanus corporis | 1 | 83 |
| **Nematodes** |  |  |
| Caenorhabditis elegans | 1 | 116 |
| Brugia malayi | 1 | 392 |
| **Cnidarians** |  |  |
| Hydra magnipapillata | 0 |  |
| Nematostella vectensis | 1 | 644 |
| **Echinodems** |  |  |
| Strongylocentrotus purpuratus | 1 | 457 |
| **Molluscs** |  |  |
| Aplysia californica | 1 | 2386 |
| **Placozoans** |  |  |
| Trichoplax adhaerens | 1 | 600 |
|  |  |  |
| **Choanoflagellates** |  |  |
| Monosiga brevicollis | 0 |  |
| Salpingoeca | 1 | 279 |
| **Filastera** |  |  |
|  |  |  |
| **Ichthyosporea** |  |  |
| Capsaspora owczarzaki | 1 | 243 |
| **Fungi** |  |  |
| **Ascomycetes** |  |  |
| Saccharomyces cerevisiae | 0 |  |
| Naumovozyma dairenensis | 0 |  |
| Ashbya gossypii | 0 |  |
| Tetrapisispora phaffii | 0 |  |
| Vanderwaltozyma polyspora | 0 |  |
| Candida albicans | 0 |  |
| Yarrowia lipolytica | 0 |  |
| **Basidiomycetes** |  |  |
| Schizophyllum commune | 0 |  |
| Cryptococcus neoformans | 0 |  |
| Postia placenta | 0 |  |
| **Microsporidians** |  |  |
| Encephalitozoon hellem | 0 |  |
| Encephalitozoon intestinalis | 0 |  |
|  |  |  |
| **Nucleariidae** |  |  |
|  |  |  |
| **Amoebozoa** |  |  |
| Entamoeba histolytica | 0 |  |
| Entamoeba dispar | 0 |  |
| Entamoeba invadens | 0 |  |
| Dictyostelium discoideum | 0 |  |
| Dictyostelium fasciculatum | 0 |  |
| Dictyostelium purpureum | 0 |  |
| Acanthamoeba castellanii | 1 | 117 |
| Physarum polycephalum | 1 | ? |
|  |  |  |
| **Total** | 17 |  |
|  |  |  |
|  |  |  |
|  |  |  |
|  | **Intron Present?** | **Intron Length (nt)** |
| **Chromalveolates** |  |  |
| **Alveolata** |  |  |
| **Apicomplexan** |  |  |
| Plasmodium falciparum | 0 |  |
| Plasmodium vivax | 0 |  |
| Toxoplasma gondii | 0 |  |
| Cryptosporidium parvum | 0 |  |
| Babesia bovis | 0 |  |
| Theileria parva | 0 |  |
| Neospora caninum | 0 |  |
|  |  |  |
| **Cilliates** |  |  |
| Paramecium tetraurelia | 1 | 23 |
| Ichthyophthirius multifiliis | 0 |  |
| **Perkinsus** |  |  |
| Perkinsus marinus | 1 | 48 |
| **Stramenopiles** |  |  |
| Thalassiosira pseudonana | 0 |  |
| Phaeodactylum tricornutum | 0 |  |
| Phytophthora infestans | 1 | 79 |
|  |  |  |
| **Rhizaria** |  |  |
| **Cercozoa** |  |  |
| Bigelowiella natans | 1 | 135 |
| **Foraminifera** |  |  |
|  |  |  |
| **Radiolaria** |  |  |
|  |  |  |
| **Hacrobia** |  |  |
| **Cryptomonads** |  |  |
| Cryptomonas paramecium | 0 |  |
| Guillardia theta | 0 |  |
| Hemiselmis andersenii | 0 |  |
| **Total** | 4 |  |
|  |  |  |
|  |  |  |
|  | **Intron Present?** | **Intron Length (nt)** |
| **Excavates** |  |  |
| **Malawimonads** |  |  |
|  |  |  |
| **Euglenozoa** |  |  |
| **Kinetoplastids** |  |  |
| Trypanosoma brucei | 0 |  |
| Trypanosoma cruzi | 0 |  |
| Leishmania major | 0 |  |
| Leishmania donovani | 0 |  |
|  |  |  |
| **Heterolobosea** |  |  |
| Naegleria gruberi | 0 |  |
| **Jakobida** |  |  |
|  |  |  |
| **Parabasalids** |  |  |
| Trichomonas vaginalis | 0 |  |
| **Fornicata** |  |  |
| **Diplomonads** |  |  |
| Giardia lamblia | 0 |  |
| Spironucleus vortens | 1 | 41 |
| **Preaxostyla** |  |  |
|  |  |  |
| **Total** | 1 |  |
