# Supplementary material for: Patterns of conservation of spliceosomal intron structures and spliceosome divergence in representatives of the diplomonad and parabasalid lineages
Source: BMC Evol Biol. 2019 Aug 2;19:162. doi: 10.1186/s12862-019-1488-y (PMC6679479; doi:10.1186/s12862-019-1488-y)
Supplement: Supplementary file 9 — Table of spliceosomal proteins found in our searches (see Methods). Numbers in red indicate that the initial psiBLAST searches had 0 hits, and HMM searches were not a part of their disqualification. Numbers in grey indicate that the protein was missing a domain from the conserved set. Sm and LSm proteins are grouped together. Hs - H. sapiens, Sc - S. cerevisiae, At - A. thaliana, Gi - G. lamblia, T - Trepomonas sp. PC1, Ss - S. salmonicida, Kb - Kipferlia bialata sp., M - Monocercomonoides sp. PA203. (PDF 57 kb) [file 12862_2019_1488_MOESM9_ESM.pdf]

Sub-complex	Protein	Hs	Sc	At	Gi	T	Ss	Kb	M
A	BUB3	1	1	1	1	1	1	1	1
A	CDK11A_CDC2L2	1	0	1	1	1	0	1	1
A	DDX17_p72	1	1	1	1	1	0	1	1
A	DDX5	1	1	1	1	1	0	1	1
A	RBM10	1	0	1	0	0	0	0	1
A	RBM39	1	0	1	0	0	0	0	0
A	SF1	1	1	1	0	0	0	0	0
A	SUGP1	1	0	1	0	0	0	0	0
A	tatSF1	1	1	1	0	0	0	0	0
Bact	CCDC12_MGC23918	1	0	1	0	0	0	0	0
Bact	PPIL2	1	0	1	0	0	0	0	0
B	Kin17	1	0	1	0	0	0	0	1
B	MFAP1	1	0	1	0	0	0	0	1
B	Smu1	1	1	1	1	1	0	1	1
B	UBL5	1	1	1	1	1	1	0	1
C2	CDK10	1	0	1	1	1	1	1	1
C2	DGCR14	1	0	1	0	0	0	0	1
C2	FRA10AC1	1	0	1	0	0	0	0	1
C2	GNB2LI_RACK1	1	1	1	1	1	1	1	1
C2	HSP70_HSPA1A	1	1	1	1	1	1	1	1
C2	NKAP	1	0	1	0	0	0	0	0
CBPProteins	CBP20	1	1	1	1	1	1	1	1
CBPProteins	CBP80	1	1	1	0	0	0	0	0
C	C19orf29_CACTIN	1	0	1	0	0	0	0	0
C	C1orf55_FLJ35382	1	0	1	0	0	0	0	0
C	C9orf78_HSPC220	1	0	1	0	0	0	0	1
C	DDX35	1	1	1	1	0	1	1	1
C	DDX41	1	1	1	0	0	0	0	1
C	GPATCH1_Q9BRR8	1	0	1	0	0	0	0	1
C	NOSIP	1	0	1	0	0	0	0	0
C	PPIG	1	0	1	1	1	1	1	1
C	PPIL3	1	0	1	1	1	1	1	1
C	PPWD1	1	1	1	0	0	0	0	1
C	WDR83	1	0	1	1	1	1	1	1
DisassemblyProteins	Ntr2	0	1	0	0	0	0	0	0
DisassemblyProteins	Prp43	1	1	1	1	0	1	1	1
DisassemblyProteins	TFIP11	1	1	1	0	0	0	1	1
EarlySplicingFactors	hPRP5	1	1	1	1	1	1	1	1
EarlySplicingFactors	TCERG1	1	1	1	0	0	0	0	1
EJC	C17orf85_ELG	1	0	0	0	0	0	0	0
EJC	DDX39B_UAP56	1	0	1	1	1	1	0	1
EJC	EIF4A3	1	1	1	1	1	1	1	1
EJC	MAGOH	1	0	1	0	0	0	1	1
EJC	PNN	1	0	1	0	0	0	0	0
EJC	RMB8A_Y14	1	0	1	1	1	0	1	1
EJC	THOC1	1	0	1	0	0	0	0	0

EJC	THOC2	1	1	1	0	0	0	0	0
EJC	THOC3	1	0	1	0	0	0	0	0
EJC	THOC4	1	0	1	0	0	0	0	1
EJC	THOC5	1	0	1	0	0	0	0	1
hnRNP	PCBP1	1	1	0	0	0	0	0	0
hnRNP	PCBP2	1	1	0	1	0	0	0	0
hnRNP	RBMX_hnRNPG	1	0	1	0	0	0	0	0
KnownSplicingFactors	CCDC130	1	1	1	0	0	0	1	1
KnownSplicingFactors	CCDC49	1	1	1	0	0	0	0	0
KnownSplicingFactors	Cwc23_DNAJA1	1	1	1	1	1	1	1	1
KnownSplicingFactors	DDX16	1	1	1	1	0	1	1	1
KnownSplicingFactors	GPKOW_T54	1	1	1	0	0	0	1	1
KnownSplicingFactors	KIAA1604	1	1	1	0	0	0	1	1
KnownSplicingFactors	NY_CO_10	1	1	1	1	1	1	1	1
KnownSplicingFactors	Prp2	1	1	1	1	0	1	1	1
KnownSplicingFactors	RNF113A	1	1	1	0	0	0	1	1
MISC	CELF_CUGBP1	1	0	1	1	1	1	1	1
MISC	CIRPB	1	0	1	0	1	0	1	1
MISC	GCFC1	1	0	1	0	0	0	0	0
MISC	KHDRBS3_SAM68	1	0	0	0	0	0	0	0
MISC	PPIL4	1	0	1	0	0	0	0	1
MISC	PPP1CA	1	1	1	1	1	1	1	1
MISC	PRMT5	1	0	1	0	0	0	0	1
MISC	RBBP6	1	0	0	0	0	0	0	0
MISC	RBM42	1	0	0	0	0	0	0	1
MISC	SAP30BP_HCNGP	1	0	1	0	0	0	0	0
MISC	ZNF207	1	0	1	0	0	0	0	0
mRNA	DDX3X	1	1	1	1	1	1	1	1
mRNA	PABPC1_PABP1	1	1	1	0	0	0	0	1
mRNA	SRRT_ASR2B	1	0	1	0	0	0	0	0
NTCPrp19	CDC5L	1	1	1	0	0	0	0	0
NTCPrp19	CRNKL1	1	1	1	0	0	0	1	0
NTCPrp19	GCIPp29	1	1	1	0	0	0	0	0
NTCPrp19	hPRP19	1	1	1	0	0	0	0	1
NTCPrp19	hSYF1	1	1	1	0	0	0	0	0
NTCPrp19	KIAA1160	1	1	1	0	0	0	1	1
NTCPrp19	Ntc20Sc	0	1	0	0	0	0	0	0
NTCPrp19	SPF27	1	1	1	0	0	0	0	1
NTCRelated	Bud31	1	1	1	0	0	0	1	1
NTCRelated	Ecm2	1	1	1	0	0	0	0	0
NTCRelated	HSPC148	1	1	1	0	0	0	0	0
NTCRelated	Prp45SKIP1	1	1	1	0	0	0	0	0
NTCRelated	Prp46PRL1	1	1	1	1	1	1	1	1
NTCRelated	RBM22	1	1	1	0	0	0	1	1
Prp19	CTNNBLI1	1	0	1	0	0	0	1	1
Prp19	HSPA8_CCAP1	1	1	1	1	1	1	1	1
Prp19rel	AQR_KIAA0560	1	1	1	0	0	0	0	0
Prp19rel	PPIL1	1	1	1	1	1	1	1	1

RES	CGI-79	1	1	0	1	1	1	1	1
RES	MGC13125	1	1	1	0	0	0	0	1
RES	SNIP1	1	1	1	1	1	1	1	1
SR	SRSF3_SRp20	1	0	0	0	0	0	0	0
SR	SRp55	1	1	0	1	1	1	1	1
SR	SRRM1_SRm160	1	0	1	0	0	0	1	1
SR	SRSF10_SRp38	1	0	1	1	1	1	1	1
SR	SRSF1_9	1	0	1	1	1	1	1	1
SR	SRSF2_SC35	1	0	1	1	1	1	1	1
SR	SRSF7_9G8	1	0	1	1	1	1	1	1
Step2Proteins	Prp16	1	1	1	1	0	1	1	1
Step2Proteins	Prp17	1	1	1	1	1	1	1	1
Step2Proteins	Prp18	1	1	1	0	0	0	1	1
Step2Proteins	Prp22	1	1	1	0	0	0	0	1
Step2Proteins	Slu7	1	1	1	0	0	0	1	1
U11U12	20k	1	0	1	0	0	0	0	0
U11U12	25k	1	0	1	0	0	0	0	0
U11U12	31k	1	0	1	0	0	0	0	0
U11U12	35kA	1	0	1	0	0	0	0	0
U11U12	35kB	1	0	1	0	0	0	0	0
U11U12	48k	1	0	1	0	0	0	0	0
U11U12	59k	1	0	1	0	0	0	0	0
U11U12	65k	1	0	1	0	0	0	0	0
U1	Luc7B1	1	1	1	0	0	0	1	1
U1	Prp39	0	1	1	0	0	0	1	1
U1	Prp40FBP11	1	1	1	0	0	0	0	1
U1	Prp42Ca	0	1	0	0	0	0	0	0
U1	Prp42Sc	0	1	0	0	0	0	0	0
U1	Snu56Sc	0	1	0	0	0	0	0	0
U1	Snu71S164	1	1	1	0	0	0	0	1
U1	TIA1	1	1	1	0	0	0	0	0
U1	U170K	1	1	1	0	0	0	0	1
U1	U1a	1	1	1	0	0	0	1	1
U1	U1c	1	1	1	0	0	0	1	1
U2rel	U2AF1_U2AF35	1	0	1	1	0	0	0	1
U2rel	U2AF2_U2AF65	1	1	1	1	0	0	1	1
U2rel	U2SURP_SR140	1	0	1	0	0	0	0	0
U2	SF3a120	1	1	1	0	0	0	1	1
U2	SF3a60	1	1	1	0	0	0	0	0
U2	SF3a66	1	1	1	1	0	0	0	1
U2	SF3b10	1	1	1	0	0	0	1	0
U2	SF3b130	1	1	1	0	0	0	1	1
U2	SF3b145	1	1	1	1	0	0	1	1
U2	SF3b14b	1	1	1	1	0	0	1	1
U2	SF3b150	1	1	1	1	0	0	1	1
U2	SF3b15.5	1	1	1	0	0	0	1	1
U2	SF3b49	1	1	1	1	0	0	1	1
U2	U2A'	1	1	1	1	1	0	1	1

U2	U2B''	1	1	1	0	0	0	1	1
U4U5U6	110K	1	1	1	0	0	0	1	1
U4U5U6	65K	1	1	1	1	1	1	1	1
U4U5U6	hPRP38	1	1	1	1	0	0	1	1
U4U5U6	hSNU23	1	1	1	1	0	0	1	1
U4U5U6	Spp381	0	1	0	0	0	0	0	0
U4U5U6	SRPK2	1	1	1	1	1	1	1	1
U4U6	15_5K	1	1	1	1	1	1	1	1
U4U6	60K	1	1	1	0	0	0	0	0
U4U6	61K	1	1	1	0	0	0	1	1
U4U6	90K	1	1	1	0	0	0	0	1
U5	100K	1	1	1	1	1	1	1	1
U5	102K	1	1	1	0	0	0	1	1
U5	116K	1	1	1	0	0	0	0	1
U5	15K	1	1	1	1	1	1	1	1
U5	200K	1	1	1	1	1	1	1	1
U5	220K Prp8	1	1	1	1	1	1	1	1
U5	52K	1	1	1	0	0	0	0	0
Sm/LSm		14	14	14	6	6	4	9	10
Totals		167	116	159	62	48	44	87	115
