# Supplementary material for: Patterns of conservation of spliceosomal intron structures and spliceosome divergence in representatives of the diplomonad and parabasalid lineages
Source: BMC Evol Biol. 2019 Aug 2;19:162. doi: 10.1186/s12862-019-1488-y (PMC6679479; doi:10.1186/s12862-019-1488-y)
Supplement: Supplementary file 10 — Primary sequence comparison of Spironucleus U2 snRNA candidates with U2 and U12 snRNAs from representative eukaryotes. This file contains U2 and U12 snRNA nucleotide alignments showing Spironucleus snRNA regions which are representative of either major (U2) or minor (U12) snRNA class. (DOCX 24 kb) [file 12862_2019_1488_MOESM10_ESM.docx]

**Additional File 10 - Primary sequence comparison of *Spironucleus* U2 snRNA candidates with U2 and U12 snRNAs from representative eukaryotes.**

Sequences for *S. vortens* and *S. salmonicida* U2 snRNAs were aligned with (**A**) U2 or (**B**) U12 snRNAs from representative eukaryotes using ClustalW2 software (Larkin *et al.* 2007). Regions of U2/12 snRNA predicted for form intermolecular base pairing with U6/U6atac or the intron branch point (BP) are indicated above the alignments. Nucleotides conserved in at least four eukaryotes are highlighted in grey. Alignments were constructed using: *S. vortens* (Sv) U2 snRNA (NCBI trace archive ti|2141663608: nucleotide positions 84-246); *S. salmonicida* (Ss) U2 snRNA (GenBank AUWU01000434:68649-68502); *Giardia lamblia* (Gl) U2 [GenBank Accession JX416862];, *Acanthamoeba castellanii* (Ac) U2 [GenBank CW933695:787-579], U12 [CW917526:369-205]; *Phytophthora spp*. (Pr) U2 [*Phythophthora ramorum* genome release V1.0 scaffold_1672:234-416], U12 (Ps) [AAQY02000248:644532-644696]; *Arabidopsis thaliana* (At) U2 [X06478:200-359], U12 [CP002684:22603122-22603295]; *Homo sapiens* (Hs) U2 [NR_002716:1-187], U12 [L43846:331-480].

**(A) U2 Alignment**

U2/U6 U2/U6

U2/U6 hII Ib Ia BP hIII

At_U2 ---ATACCTTTCTCGGCCTTT--TGGCTAAGA-TCAAGTGTAGTATCTGTTCTTATCAGT 54

Hs_U2 ---AT-CGCTTCTCGGCCTTT--TGGCTAAGA-TCAAGTGTAGTATCTGTTCTTATCAGT 53

Ac_U2 -ACAT---CTTCTCGGCCCAAG-TGGCTAAGA-TCATGTGAAGTATCTGTTCTTATCAGC 54

Pr_U2 ---AC---CTTCTCGGCCTTT--TGGCTAAGA-TCAAGTGTAGTATCTGTTCTAATCAGT 51

Gl_U2 TAAAA-----TCAGAGTC------GGCTTCGACTTTAGTGTAGTTACTGTT-TCGTCGGC 48

Sv_U2 -TCAG---CATCACGGAAGTGATTTGCTCAGA-TCAAGTGTAGTACAAGTTTCGGCCCTG 55

Ss_U2 TCTAT---CATT-CAGAAGTGACATGCTTAGA-TCAACTGTAGTACAAGTTTATACCTTA 55

* * * *** ** * ** *** *** *

Sm site

At_U2 TTAATATCTGAT----ATGTGGGCCATCGGCCCACACGATATTAACTCTATTTTTTAAGG 110

Hs_U2 TTAATATCTGAT----ACGTCCTCTATCCGAGGACAATATATTAAATGGATTTTTGGAGC 109

Ac_U2 TTAATCTCTGGTAGTGAGGCCTCCTGTGCCTCACCTCAAGGTTAGACTTATTTTTCTTGT 114

Pr_U2 GTGAAAACTGGTTCCGACGTTTTTCGTTGGTCTTTT-----TCACATTCATTTTT----- 101

Gl_U2 TTAACCGCCGAT-----------CCAC---------------TACATGCA---------A 73

Sv_U2 GTAAAGCAGGGCCTTCCGGTACGCC-GGAGCT-TC-------CACTTTTATCATCCGGTC 106

Ss_U2 GTGAAATAAGGTATCCAGATATTTCTGGTATTATC-------TTTTTTTATAACT---TC 105

* * * *

At_U2 GAGAAAGCCCGT-TAAGAT--------AGCT-TGCT-------------------AT--C 139

Hs_U2 AGGGAGATGGAA-TAGGA----------GCT-TGCTCCGTCCACTCCACGCATCGAC--C 155

Ac_U2 TGGGC-TCCTGG-CACCATGCCCTTCCAGCTATGCTGTGGGCAGTCCAGAGAGCAGTGAT 172

Pr_U2 -GGGCATCCCGA-TGTCGCGC------AGCT-TGCTGTGCGAGGTC---GGGGCGGTTTC 149

Gl_U2 GGGGCAGCCGGGCTGTGAGGC------AGCT--GCC-------------AGGATGGT--C 110

Sv_U2 TGGGCCACTCCCTCGGGACACCG----GGATTCGTC-----------------------C 139

Ss_U2 AGGAAAGTTTCCGCGAAAG-------------CATC-----------------------T 129

*

At_U2 TGG---------GCTTT-------CGCGA-GTCGCCCA---- 160

Hs_U2 TGGTATTGCAGTACCTC-------CAGGA-ACGGTGCACC-- 187

Ac_U2 CAG---CTTTGTACTGCACCACCCTGCAAAGTTCTTCAAAT- 210

Pr_U2 CGGGGGCTTT-CACCTCTCC--CCCGCGAGGC----CAAC-- 182

Gl_U2 CTG----CCCTTGTCCCGGC------TGGCGCCGTCCACCTT 142

Sv_U2 CGGGCATTCATGGCTCC-----------AGGCCGC------- 163

Ss_U2 TTGGGAATCA--ACCT--------------GCTGT------- 148

*

**(B) U12 Alignment**

U12/U6atac U12/U6atac

Ib Ia BP hIII

Hs_U12 ATGCCTTAA-ACT-TATGAGTAAGGAAAATAACGA-TTCGGGGTGA--CG--C-CCGAAT 52

Ac_U12 --GCCTTAA-ACT-AATGAGTAAGGAAAATACCGC-ACCGGT-TGA--CA--C-CGGTGT 49

Ps_U12 --GGCTTAA-ACTCAATGAGTAAGGAAACTAACGC-CCTACC-TGA--CA--AGTAGGGC 51

At_U12 --GCCTTAA-ACT-AATGAGTAAGGAAAACAAAGCGTCCGGTGAGAACCGGTCGCGGCGC 56

Gl_U2 ------TAA-AATCA--GAGTCGG-----CTTCGACTTTAGTGTAGT-------TACTGT 39

Sv_U2 -----TCAGCATCACGGAAGTGAT--TTGCTCAGA-TCAAGTGTAGTACA-----AGTTT 47

Ss_U2 ----TCTATCATT-CAGAAGTGAC--ATGCTTAGA-TCAACTGTAGTACA-----AGTTT 47

* * *** *

Sm site

Hs_U12 C--CTCAC--------TGCTAA--TGTGAGA--CGAATTTTTGAGCGGG-TAAA--GGTC 95

Ac_U12 T--ATGGCGTGAACCGCGTTCA--CGTCGGCTTCCAATTTCTG-GCGGGCTACA--GGCC 102

Ps_U12 TGCCTGTTGGATCGAGTGATC---CATGGGTTTCTAATATTTGACGGAGGTGGAATGGCC 108

At_U12 TAATCGTAAAACACAAAATTGG--CGCAA-----TAATTTATGGAGGGGTTATA--GGCT 107

Gl_U2 T--TCGTCG--------GCTTAACCGCCGAT--CCACTACATGCAAGGGGCAGCCGGGCT 87

Sv_U2 CGGCCCTGG----------TAAAGCAGGGCCTTCCGGTACGCC--GGAGCT-TC------ 88

Ss_U2 ATACCTTAG----------TGAAATAAGGTATCCAGATATTTCT-GGTATTATC------ 90

* * *

Hs_U12 ---GCCCTCAAG--GTGACC------CGCCTACTTT---GCGGGA----------TG-CC 130

Ac_U12 TCGGCCCTCTTGTAGTGACCTG----CGCCTACTTTCGCGCGGGA----------TG-CT 147

Ps_U12 ---GTCATTTTA--GGAACC------CGC-TACTTT----TAGGG----------TGACT 142

At_U12 --GGCCGATGTGT--TGACGC-----TGCTTACTTTT--GCAGAA----------CTCAC 146

Gl_U2 --------------GTGAGG------CAGCTGC-------CAGGA----------TGGTC 110

Sv_U2 ----CACTTTTA---TCATCCGGTCTGGGCCACTCCC--TCGGGACACCGGGATTCGTCC 139

Ss_U2 ----TTTTTTTA---TAACT---TCAGGAAAGTTTCC--GCGAAAG---------CATCT 129

*

Hs_U12 TGGGAGTTG--CGATCTGC--CCG-------- 150

Ac_U12 C--GAGTTGGCTGGCCT----CCG-------- 165

Ps_U12 AAAAAGGGGGCCGGTCC----CCGCCC----- 165

At_U12 CTCGTGCGGGCCTCCCTACACCCATCCC---- 174

Gl_U2 CTGCCCTTGTCCCGGCTGGCGCCGTCCACCTT 142

Sv_U2 CGGGCATTCAT-GGCTCCAGGCCGC------- 163

Ss_U2 TTGGGAATCA---ACCT---GCTGT------- 148

*
